# CSAD inhibits excessive inflammation during viral infections through the NF-κB signaling pathway

**DOI:** 10.1128/jvi.00706-25

**Published:** 2025-09-15

**Authors:** Yufan Zhang, Xiaotian Yang, Yun Chen, Junnan Shi, Kai Zhou, Li Zhu, Jing Wang, Wei Jiang, Dahui Zhao, Lipeng Gan, Sanguo Ren, Min Fang

**Affiliations:** 1 CAS Key Laboratory of Pathogenic Microbiology and Immunology, Institute of Microbiology, Chinese Academy of Sciences 85387, Beijing, China; 2 University of Chinese Academy of Sciences 74519, Beijing, China; 3 School of Life Sciences, Henan University 12411, Kaifeng, Henan, China; 4 Henan Key Laboratory of Synthetic Biology and Biomanufacturing, Henan University 12411, Kaifeng, Henan, China; 5 School of Basic Medical Sciences, Wenzhou Medical University 26453, Wenzhou, China; Emory University School of Medicine, Atlanta, Georgia, USA

**Keywords:** CSAD, host factor, NF-κB signaling pathway, inflammatory responses, viral infection

## Abstract

Studies have shown that 129 mice generate more severe inflammatory responses than C57BL/6 (B6) mice shortly after influenza virus infection. However, the mechanisms for regulation of the magnitudes and kinetics of innate immune responses in different hosts remain to be fully understood. In this study, we found that cysteine sulfinic acid decarboxylase (CSAD), an essential enzyme in the taurine synthesis pathway, inhibits excessive inflammation after viral infection. CSAD KO mice in B6 background initiate stronger inflammatory responses and are more vulnerable to viral infections, exhibiting a phenotype resembling 129 mice. Interestingly, CSAD is differentially expressed in 129 and B6 mice; CSAD is highly expressed in B6 mice than in 129 mice. CSAD inhibits the NF-κB signaling pathway during various virus infections and stimulations. CSAD directly interacts with IKKα at the kinase domain, thus limiting the fast and strong phosphorylation of IKKα and then the downstream signaling pathway. Therefore, CSAD is a novel regulator to fine-tune the intensity of the NF-κB signaling pathway.

**IMPORTANCE:**

The mechanisms by which host factors regulate the intensity of innate immune responses are important because excessive inflammatory response can be harmful to the host. CSAD, an enzyme in the taurine synthesis pathway, inhibits excessive inflammatory responses after viral infection or stimulation by interacting with IKKα of the NF-κB signaling pathway, thus limiting the downstream activation of signaling and reducing the cytokine and chemokine gene expression. Our studies reveal for the first time that CSAD plays an important role in regulating innate immune responses, adding novel regulators to the complex networks of the NF-κB signaling pathway. Furthermore, our results also help us further understand the variations in the innate immune responses among individuals and provide a novel perspective for the development of new drugs or therapies for infectious diseases.

## INTRODUCTION

Innate immune responses provide the first line against pathogenic invasions such as viruses and bacteria and stimulate the later responses of adaptive immunity via specific cell types, cytokines, chemokines, etc (1) In response to the stimuli that can be harmful conditions to the hosts, such as virus infection, physical injury, ischemic injury, and exposure to toxins, the inflammatory response, which involves innate and the following adaptive immune response, can be activated (2–6). However, the strength of the inflammatory responses should be appropriately regulated so that it can clear the pathogens and help the host recover from infectious diseases. Strong inflammatory responses can result in excessive inflammation and an upregulation of proinflammatory cytokine production (7), which is called a “cytokine storm,” and it can be detected usually in several pulmonary infectious diseases, such as influenza (8) or COVID-19 disease (9).

Of note, plenty of host signaling pathways are implicated in the regulation of inflammatory responses in innate immunity during pathogenic stimulation, including the NF-κB signaling pathway (10–12). The nuclear factor κB (NF-κB) signaling pathway can be stimulated by external stimuli. It regulates the expression of hundreds of genes related to many physiological processes, like inflammatory responses, cell proliferation, cell differentiation, cancer, autophagy, and apoptosis (13, 14). It includes the canonical pathway and the noncanonical pathway. These two pathways do not work independently but have an impact on each other after activation of the NF-κB pathway (15). Activation of the NF-κB canonical pathway relies on phosphorylation and degradation of inhibitor of κB (IκBs), predominantly IκBα, and the nuclear translocation of NF-κB complex, especially p50/Rel A (also called p65) dimer. The phosphorylation and degradation of IκB are mediated by the IκB kinase (IKK) complex (16). In the noncanonical pathway, the function of IκB is replaced by the complex of p52 and p100. p100 can be phosphorylated by IKKα and then partially degraded. This process generated p52/RelB complexes, which are translocated into nuclear and modulate gene expression (17). Among all these components, the IκB kinase (IKK) complex, which is a trimeric complex composed of IKKα (IKK1), IKKβ (IKK2), and IKKγ (also called NEMO), is a core component of the NF-κB signaling pathway, especially the canonical one. IKKα and β have a similar structure (50% sequence identity), including four main domains: N-terminal kinase domain, which contains key serine residues in the T loop that is essential in phosphorylation; C-terminal NEMO-binding domain (NBD), that can mediate the binding of NEMO; Leucine Zipper (LZ) domain and Helix-Loop-Helix (HLH) domain, which are important for dimerization of IKKα and IKKβ (18).

The regulation of the NF-κB signaling pathway is extremely complicated and important because the strengths of the inflammation responses play an important role in the outcome during virus infection. NF-κB signaling can be modulated by a lot of factors from the host or pathogen. Viral proteins have been reported to modulate the NF-κB signaling pathway; for instance, during influenza A virus (IAV) infection, NS1 interacts with IKKα/IKKβ with the C-terminal effector domain and inhibits kinase activation (19). The polymerase of hepatitis B virus (HBV) and the ankyrin-repeat proteins of poxvirus can inhibit IκB functions (20). Meanwhile, some viral proteins can activate the NF-κB signaling pathway. Influenza virus protein PB1-F2 interacts with IKKβ to enhance its phosphorylation (21); HIV-1 virus protein Vpr and Tat by interacting with IKKα/IKKβ and IκB, respectively (22, 23). In addition, host factors also regulate the NF-κB signaling pathway. Our previous work has shown that UDP-GalNAc transferase 3 (GALNT 3) inhibits the NF-κB signaling pathway by preventing the translocation of phosphorylated p65 into the nucleus (24). Other researchers also found that loss of p53 enhances IKKβ catalytic activity via O-linked beta-N-acetyl glucosamine modification (25). Additionally, studies show that IKK-interacting protein (IKIP) inhibits the phosphorylation of IKKα/β, such as a 15-aa peptide from mouse IKIP that can disrupt the association of IKKβ and NEMO (26).

In addition to the regulation of signaling pathways, different individuals exhibit different immune response strengths. Studies showed that different mice strains display different levels of interferon secretion after virus infection (27). Our previous research also demonstrated that during high-dose IAV infection, the susceptible 129 mice exhibit severe lung injury and quick production of inflammatory cytokines compared with the more resistant B6 mice (28). A total of 287 human host factor genes identified by genome-wide RNAi screen influence influenza virus replication (29). Single-nucleotide polymorphism (SNP) of human host factors between individuals causes differences in responses during virus infection (30). Recently, the variant MX1 gene in different individuals, which encodes myxovirus resistance protein A (MXA) that plays a crucial role in IAV infection control, is confirmed as a piece of genetic evidence in controlling zoonotic IAV infection (31). Therefore, it is important to consider the host factors in different individuals, which engage in various physiological processes including signaling pathways in innate immunity related to the differences in strength of immune responses.

CSAD is an enzyme that is involved in the second step of the taurine synthesis pathway (32, 33). Taurine is one of the most abundant amino acids found in organisms across eukaryotic phyla (34–36) and plays an important role in several basic biological processes. Taurine deficiency during early life causes functional impairments in the skeletal muscle, eye, and the central nervous system (37, 38). CSAD is highly conserved in mammals. In humans, CSAD is widely expressed in different tissues and organs. Previous studies show that CSAD ameliorates lipid accumulation in high-fat diet-fed mice, and the effect of CSAD on lipid accumulation might be independent of the taurine pathway (39). A genome-wide association study identifying variants in *CSAD/lnc-ITGB7-1* associates with susceptibility to fulminant type 1 diabetes (40). So far, most reports on CSAD function have focused on metabolism and mammalian development, while its role in the immune response has not been explored. In this research, we explored the role of CSAD during viral infections.

## RESULTS

### CSAD prevents excessive inflammation in B6 mice

**Fig 1:**
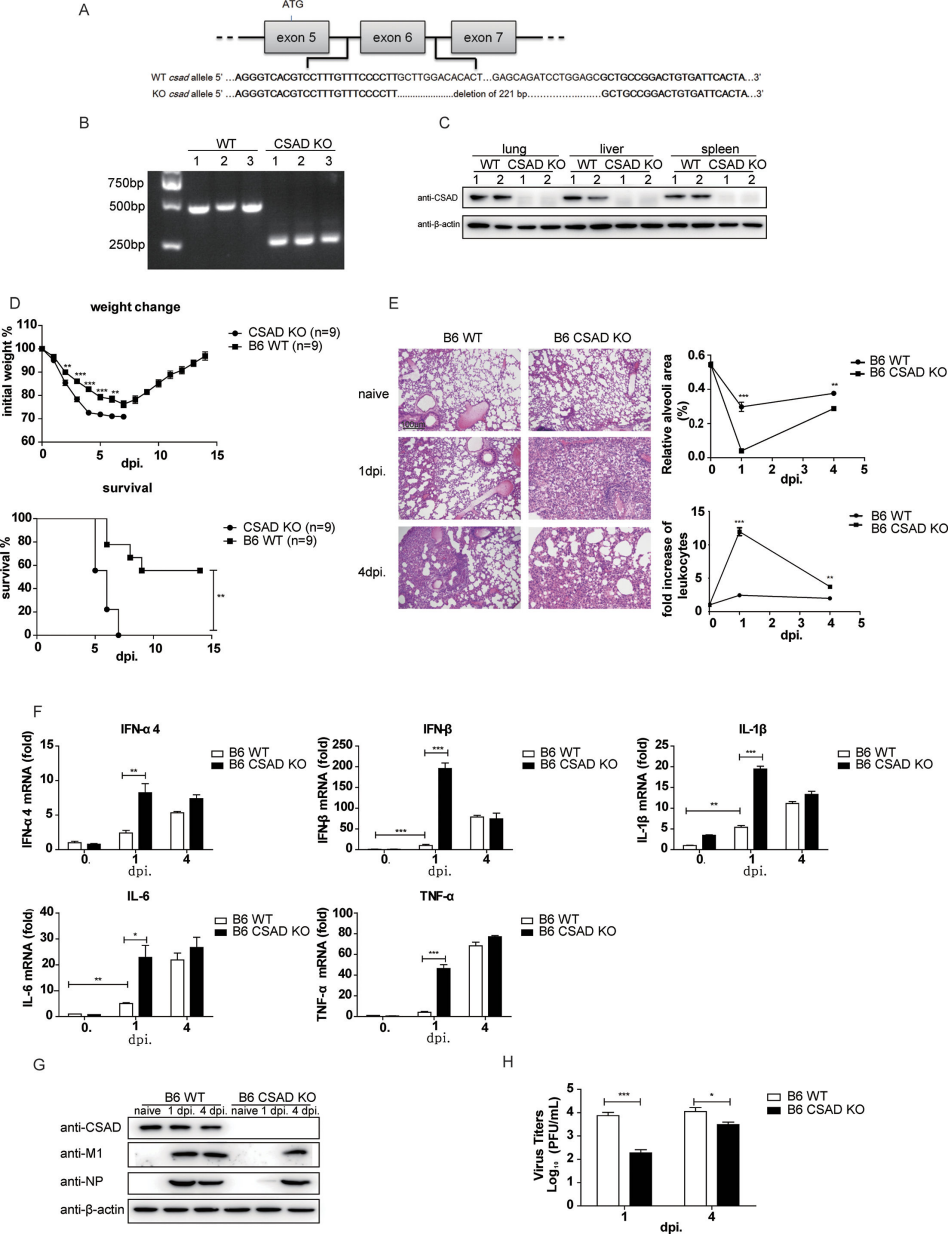
CSAD KO mice show excessive early inflammatory responses and high mortality after IAV infection. (**A**) Construction of CSAD KO mice. The exon 6 in the gene allele of CSAD was deleted of 221 bp. (**B**) Mice tails were collected and lysed with lysis buffer, the supernatants were collected and genome CSAD was amplified by PCR, and detected by agarose gel electrophoresis assay. (**C**) The indicated mice were sacrificed, and organs were collected and lysed by lysis buffer, and then Western blot was used to detect CSAD levels in different mice organs. (**D–H**) CSAD KO mice and WT B6 mice were infected with 1 × 10^6^ pfu PR8 virus. (**D**) Weight change and survival rate. Weight loss and survival rates were monitored. (**E**) Haematoxylin-eosin (H&E) staining of the lung sections at 1 dpi and 4 dpi (original magnification ×10; scale bars, 100 µm). (left and statistics analysis of relative alveoli area and fold change of leukocyte infiltrates (right) by ImageJ software. (**F**) The transcripts of indicated genes were detected by qRT-PCR in pulmonary homogenates at the indicated time points. (**G**) Pulmonary homogenates were prepared to measure the levels of CSAD, viral protein M1 and NP by Western blot assay. (**H**) Viral titers of pulmonary homogenates in B6 and CSAD KO mice. Pulmonary homogenates were prepared to determine viral titers by plaque assay in MDCK cells. The data correspond to the means ± SEM for at least two independent experiments. *P < 0.05; **P < 0.01; *** *P* < 0.001.

CSAD, an important enzyme in the taurine synthesis pathway, has important functions in metabolism (32, 33); however, whether CSAD plays any role in immune responses has not been explored. To study the function of CSAD in immunity, we constructed CSAD knockout mice in the B6 genetic background. A 221 bp fragment was depleted in exon 6 of the CSAD gene (Gene ID: 246277) allele (Fig. 1A). The KO mice were confirmed by PCR (Fig. 1B), and CSAD expression was aborted in various organs including the lung, liver, and spleen in the KO mice, as detected by Western blot, demonstrating that CSAD KO mice were successfully constructed (Fig. 1C). The CSAD KO mice were healthy and able to give birth normally by feeding with 0.05% taurine in their drinking water as taurine is a key element in many diverse processes including the development of the brain, retina, and the immune system (41).

Next, 1 × 10^6^ pfu influenza A/Puerto Rico/8/34 (PR8) was used to infect these mice, and then weight loss and survival rates were detected. Of note, B6 CSAD KO mice were more vulnerable to virus infection as their weight loss reduced much more obviously and their survival rate was much lower than that of B6 WT mice (Fig. 1D). According to previous research, severe inflammation is an important contributing factor that leads to inflammatory cell infiltration and cytokine storm, causing high morbidity and mortality in mice after virus infection (42). WT B6 mice and CSAD KO mice infected with PR8 were sacrificed at 1 day or 4 days post-infection (dpi), and hematoxylin-eosin (H&E) was used to stain the lung section to detect the inflammatory responses. We found that at the early stage (1 dpi) of PR8 infection, CSAD KO mice exhibited more inflammatory infiltrates than those in B6 mice. However, the inflammation in CSAD KO mice was similar to that in B6 mice at 4 dpi. (Fig. 1E, left panel). Consistently, in CSAD KO mice, the relative alveoli area was reduced significantly at 1 dpi., and the infiltration of leukocytes was increased compared with that of B6 mice (Fig. 1E, right panel). Then, we measured the mRNA level of several cytokine genes in B6 and CSAD KO mice, which were infected with 1 × 10^6^ pfu PR8 and sacrificed at 1 dpi or 4 dpi. The expression levels of IFN-α4, IFN-β, TNF-α, IL-1β, and IL-6 mRNA all increased in WT B6 mice and CSAD KO mice after infection. However, the fold increase in cytokine gene expressions in CSAD KO mice was significantly higher than that of the B6 mice at 1 dpi. There were no differences in cytokine expression between the CSAD KO mice and B6 mice at 4 dpi (Fig. 1F), which was consistent with the early heavy inflammation in the lung by H&E staining. Thus, CSAD KO mice mounted stronger and earlier inflammatory responses after IAV infection.

Then, we detected viral replication capacity in B6 and CSAD KO mice by Western blot assay, and we found that PR8 replication was suppressed early at 1 dpi in CSAD KO mice as the PR8 viral proteins M1 and NP were rarely detected in CSAD KO mice at 1 dpi. But at 4 dpi, virus replication was similar between B6 and CSAD KO mice (Fig. 1G). In addition, virus titers in mice lung homogenates collected from CSAD KO mice and B6 mice after PR8 infection at different time points were measured by plaque assay, and results also indicated that virus replication was significantly repressed in CSAD KO mice at 1 dpi (Fig. 1H). The suppression of early PR8 replication in CSAD KO mice might be caused by the strong inflammatory reaction.

Our above results demonstrated that CSAD KO mice mounted stronger and earlier inflammation responses after IAV infection. The CSAD KO mice were stopped feeding with taurine after weaning (3–4 weeks of age), and all the mice were not treated with taurine during the experimental process. Because taurine plays an important role in many diverse processes, including gut microbiota and metabolism (43, 44). We further determined whether taurine plays any role in the phenotype of the KO mice after IAV infection. Hence, a group of CSAD KO mice was continually treated with taurine daily after weaning. A group of age-matched WT mice was treated with taurine 10 days before infection. Other groups of CSAD KO mice and WT mice did not receive taurine treatment. All the groups of mice were infected with 1 × 10^6^ pfu PR8. In the taurine groups, CSAD KO or WT mice were continually treated with taurine till 15 dpi. The weight change and survival rates in all four groups of mice were monitored. We found that there were no significant differences between the taurine-treated or not treated KO mice and taurine-treated or not treated WT mice. However, the weight loss and mortality rates were all higher in the KO mice groups compared with the WT mice groups (Fig. S1). These results indicated that the absence of CSAD, rather than taurine, plays a crucial role in the phenotype of the KO mice after IAV infection. Thus, our data indicate that CSAD prevents excessive inflammation in B6 mice after IAV infection. Knockout of CSAD results in excessive early inflammation and high mortality in IAV-infected mice.

### CSAD is differentially expressed in different mouse strains.

**Fig 2:**
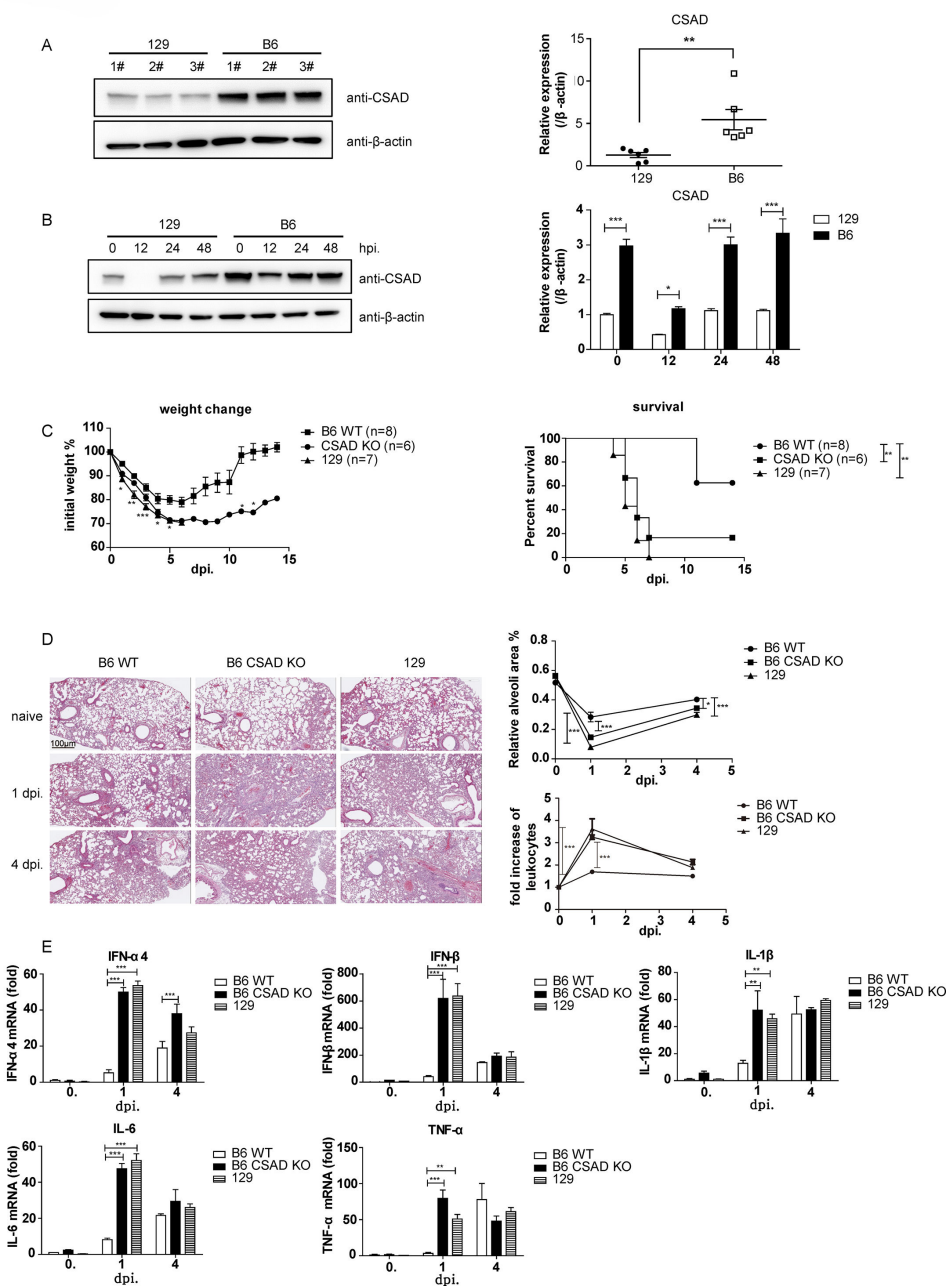
CSAD is differentially expressed in mouse strains. (**A**) CSAD expression level in 129 and B6 mice. 129 and B6 mice were sacrificed, and pulmonary homogenates were prepared to measure the levels of CSAD by Western blot assay. (**B**) CSAD expression level in 129 and B6 mice after PR8 infection. 129 and B6 mice were infected with 1 × 10^6^ pfu PR8. The mice were sacrificed, and pulmonary homogenates were prepared to measure the levels of CSAD by Western blot assay at 12, 24, and 48 hpi. Data are representative of at least five similar independent experiments. (**C–E**) 129, CSAD KO, and B6 mice were infected with 1 × 10^6^ pfu PR8 virus. (**C**) Weight change and survival rate. Weight loss and survival rates were monitored daily after PR8 infection. Data represent one experiment from at least two similar independent experiments. (**D**) H&E staining of the lung sections at 1 dpi and 4 dpi (original magnification ×10; scale bars, 100 µm). (left) and statistics analysis of relative alveoli area and fold change of leukocyte infiltrates by ImageJ software (right). (**E**) The transcripts of indicated genes were detected by qRT-PCR in pulmonary homogenates at the indicated time points. Data are representative of at least five similar independent experiments. Data points indicate means ± SEM. *P < 0.05; **P < 0.01; *** *P* < 0.001.

CSAD is highly conserved in mammals. In humans, CSAD is widely expressed in different tissues and organs, with relatively high expression in the liver, lung, breast, and adipose tissue (Fig. S2A). NCBI transcription data also showed that CSAD is widely expressed in mice and relatively highly expressed in several organs, including adult liver, kidney, and lung (Fig. S2B). Our previous studies and other studies showed that 129 mice mount earlier and stronger inflammatory responses than B6 mice after IAV infection, leading to excessive lung inflammation and high mortality (27, 28). CSAD KO mice mounted earlier and stronger inflammatory responses than B6 mice after PR8 infection, which resembles the inflammatory responses in 129 mice. Hence, we detected the expression level of CSAD in 129 and B6 mice to investigate whether the expression level of CSAD is different in the two mouse strains. Western blot was used in this experiment to detect CSAD levels in mice pulmonary homogenates, and we found that CSAD was highly expressed in B6 mice compared with that in 129 mice (Fig. 2A). When these mice were infected with PR8, CSAD levels decreased at 12 hours post-infection (hpi) and recovered at 24 hpi. However, at each time point, the expression of CSAD in B6 mice was higher than that in 129 mice (Fig. 2B).

Next, we compared the phenotype of CSAD KO mice and 129 mice after IAV infection. The 129, CSAD KO, or B6 mice were infected with 1 × 10^6^ pfu PR8. As shown in Fig. 2C, CSAD KO mice and 129 mice were more vulnerable to virus infection as their weight loss reduced much more obviously and their survival rates were much lower than that of B6 WT mice. Furthermore, at the early stage (1 dpi) of PR8 infection, CSAD KO mice and 129 mice exhibited more inflammatory infiltrates than those in B6 mice. The relative alveoli areas were reduced significantly, and the infiltration of leukocytes was increased in both CSAD KO mice and 129 mice compared with that of B6 mice at 1 dpi. However, the inflammation in these three mouse strains was similar at 4 dpi (Fig. 2D). Then, we measured the mRNA level of several cytokine genes. The expression levels of IFN-α4, IFN-β, TNF-α, IL-1β, and IL-6 mRNA all increased in mice after PR8 infection. Consistently, the fold increase of cytokine gene expressions in CSAD KO and 129 mice was significantly higher than that of the B6 mice at 1 dpi, and there were no significant differences in cytokine expression levels in these three mouse strains at 4 dpi (Fig. 2E), which was consistent with the early heavy inflammation in the lungs of CSAD KO and 129 mice by H&E staining. These data demonstrated that CSAD is differentially expressed in 129 and B6 mice. The excessive early inflammation in CSAD KO mice after PR8 infection resembles the phenotype of 129 mice.

### CSAD inhibits the NF-κB signaling pathway by interacting with IKKΑ

**Fig 3:**
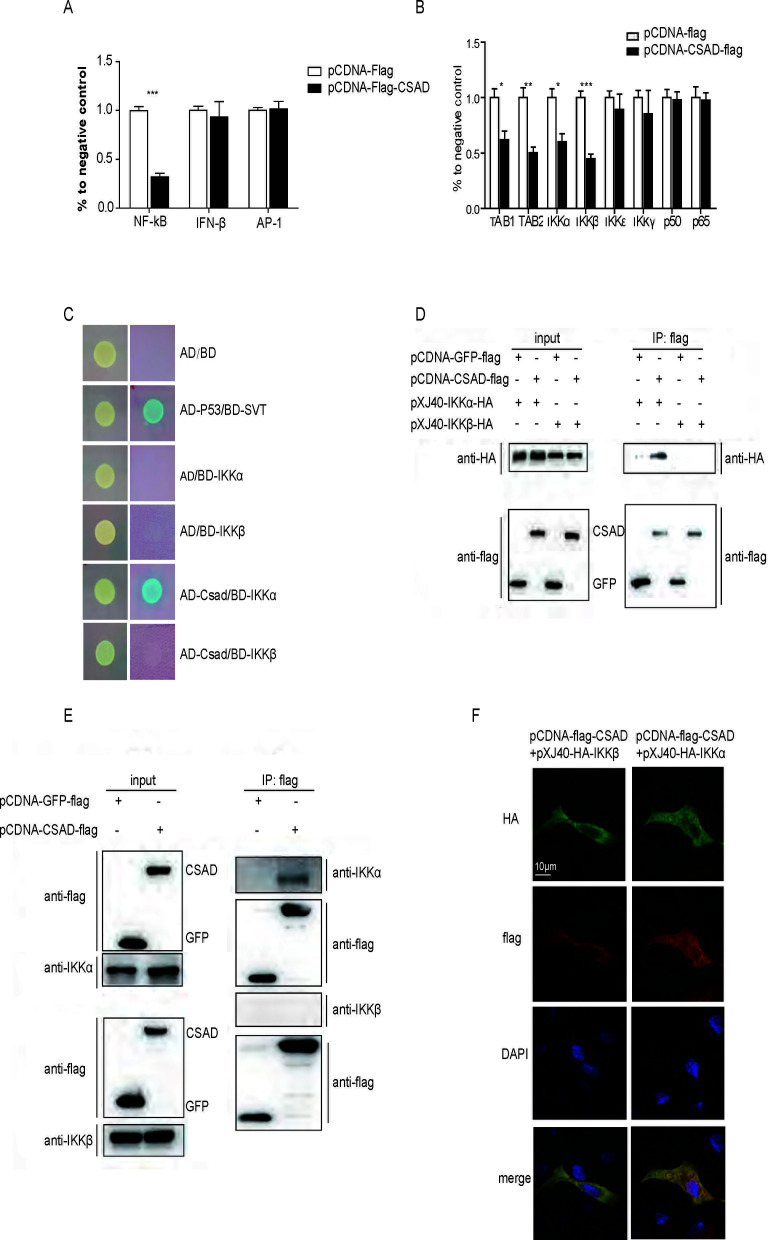
CSAD limits the NF-κB signaling pathway by interacting with IKKα. (**A**) CSAD inhibits NF-κB and RIG-I signaling pathways. 293T cells were transfected with the indicated plasmids pRL-TK, pCDNA-CSAD-Flag, and pGL3-luc-RIG I/NF-κB/AP1/IFN-β, respectively. The relative luciferase activities were measured. Each set of assays was performed in triplicate. (**B**) Identification of the adapter protein. 293T cells were transfected with pRL-TK, pCDNA-Flag/Csad and pGL3-luc-NF-κB, and TAB1/TAB2/IKKα/IKKβ/IKKγ/p50/p65. The relative luciferase activities were measured. Data are presented as the mean ± SEM from three independent experiments. *, *P* < 0.05; **, *P* < 0.01; ***, *P* < 0.001. (**C**) Interaction between CSAD and IKKα/β by yeast two-hybrid assay. pGBKT7-IKKα/β and preys pGADT7-CSAD were co-transformed in the Y2H yeast strain. Transformants were selected for growth in -T-L plates and then transferred to -T-L (left) and QDO + A + X plates (right). (**D**) Co-immunoprecipitation between CSAD and IKKα/β. 293T cells were transfected with pCDNA-CSAD-Flag or pCDNA-GFP-Flag and pXJ40-HA-IKKα or pXJ40-HA-IKKβ. The cell lysates were incubated with anti-flag affinity gel and analyzed by Western blot with the indicated antibodies. (**E**) Co-immunoprecipitation between CSAD and endogenous IKKα/β. 293T cells were transfected with pCDNA-CSAD-Flag or pCDNA-GFP-Flag. The cell lysates were incubated with anti-flag affinity gel and analyzed by Western blot with the indicated antibodies. (**F**) Colocalization of CSAD and IKKα or IKKβ. HeLa cells were cotransfected with pCDNA-flag-CSAD and pXJ40-HA-IKKα or pXJ40-HA IKKβ for 24 hours and then fixed, permeabilized, and stained for HA (green) or flag (red). Yellow indicates overlap. Data are representative of one experiment from at least three similar independent experiments.

Our previous data demonstrated that B6 and CSAD KO mice generate different inflammatory responses early after IAV infection, and CSAD is differentially expressed in 129 and B6 mice, which exhibit different strengths of inflammatory responses. Thus, we investigated whether CSAD plays any role in the antiviral innate signaling pathways. Luciferase assay was used to find the signaling pathways that might be affected by CSAD. 293T cells were co-transfected with pGL3-luc- NF-κB/AP1/IFN-β, and pCDNA-Flag or pCDNA-Flag-CSAD. We found that co-transfection with CSAD resulted in significant inhibition of NF-κB activation (Fig. 3A). Next, we further investigated which adapter protein in the NF-κB pathway might be influenced by CSAD. 293T cells were co-transfected with pCDNA-Flag or pCDNA-CSAD-Flag, pGL3-luc-NF-κB, and TAB1, TAB2, IKKα, IKKβ, IKKγ, p50, or p65, respectively. NF-κB activation was inhibited when cells were co-transfected with CSAD and TAB1, TAB2, IKKα, or IKKβ, but not in other groups (Fig. 3B). In the NF-κB signaling pathway, the IKK complex is downstream of TAB1/2; therefore, CSAD might interact with IKKα/β.

We next focused on the influence of CSAD on IKK α/β in the NF-κB signaling pathway. A yeast two-hybrid system was used to detect which IKK protein interacts with CSAD. pGBKT7(pBD)-IKKα/β or empty vector pGBKT7(pBD-T7) was transformed into the Y2H Gold yeast strain, and pGADT7(pAD)-CSAD or empty vector pGADT7(pAD-T7) was transformed into the Y2H Gold yeast strain. The interaction between p53 and SV40 large T-antigen (p53/T-antigen) was used as a positive control. pAD-T7 and pBD-T7 and pAD-T7 and pBD-IKKα/β were employed as negative controls. As shown in Fig. 3C, CSAD interacted with IKKα, but not IKKβ. We further used coimmunoprecipitation to confirm that CSAD interacts with IKKα/β. 293T cells were co-transfected with pCDNA-FLAG-CSAD/-GFP and pXJ40-HA-IKKα or IKKβ. Anti-flag affinity gel was incubated with the cell lysis supernatant, and Western blot was used to detect the interaction between CSAD and external IKKα/β. We found that exogenous CSAD interacted with exogenous IKKα, but not IKKβ (Fig. 3D). Next, we further detected whether CSAD interacts with endogenous IKKα or IKKβ. We then transfected 293T cells with pCDNA-Flag-CSAD/-GFP, anti-flag affinity gel was incubated with the cell lysis supernatant, and the interaction between CSAD and internal IKKα/β was detected by Western blot. CSAD only interacted with endogenous IKKα but not IKKβ (Fig. 3E). Further, we detected the colocalization of CSAD and IKKα or IKKβ. The indicated plasmids encoding CSAD and IKKα or IKKβ were co-transfected into HeLa cells, and the colocalization of proteins was detected by using a microscope. As shown in Fig. 3F, CSAD showed much stronger colocalization with IKKα than with IKKβ. These data demonstrated that CSAD interacts with IKKα.

### CSAD limits early phosphorylation of IKKα

**Fig 4:**
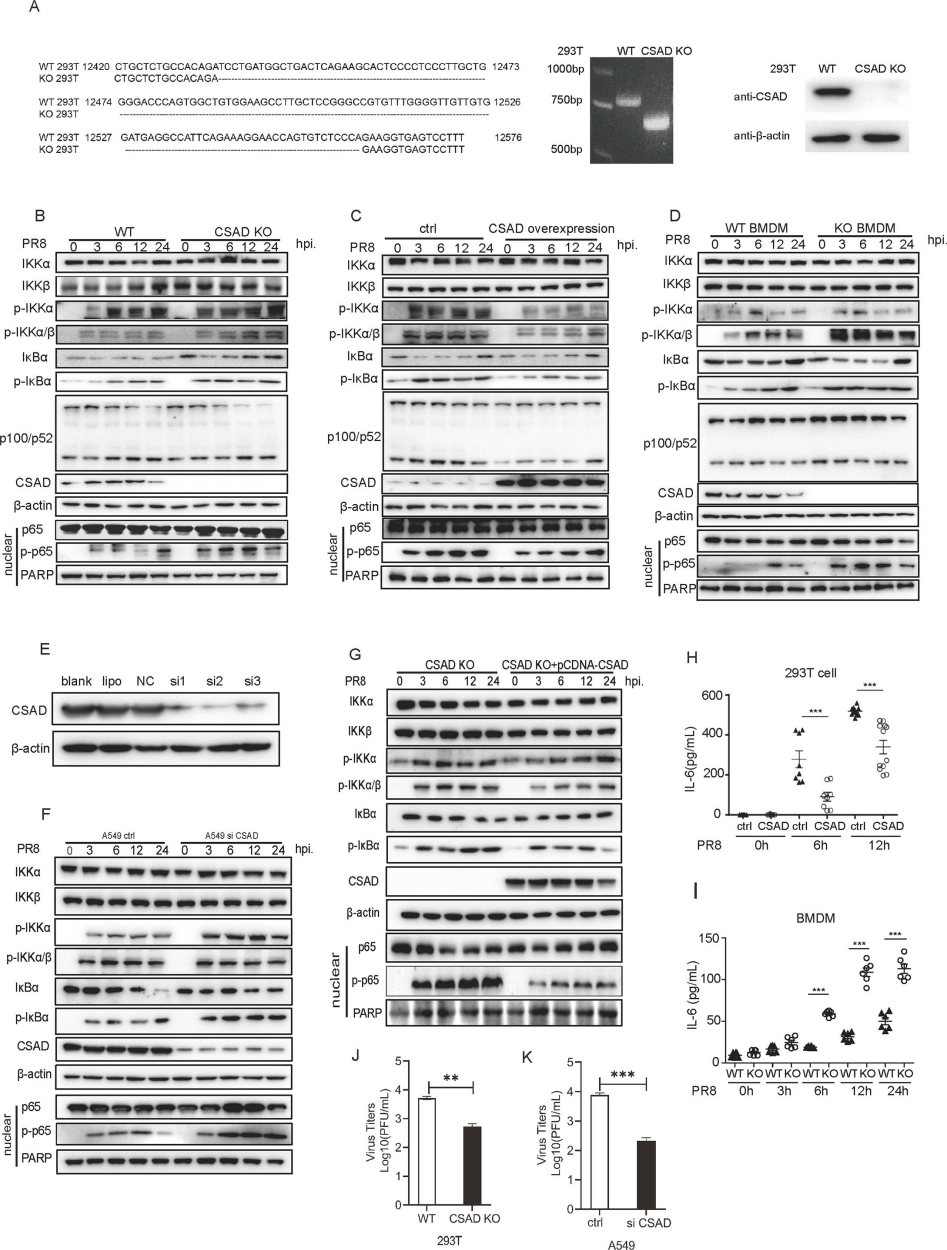
CSAD limits early phosphorylation of IKKα and downstream activation of the NF-κB signaling pathway. (**A**) Construction of the 293T CSAD KO cell. A monoclonal KO cell line was obtained with a 128 bp deletion at exon 4 of the CSAD gene. PCR and Western blot assay were used to verify the KO efficiency of CSAD in cells, respectively. (**B and C**) CSAD KO 293T cells and CSAD overexpressing 293T cells were infected with PR8 at an MOI = 1 The levels of IKKα, IKKβ, p-IKKα, p-IKKα/β, IκB-α, p-IκB-α p100/p52, IRF3, p-IRF3, CSAD, and β-actin in total cell lysates; p65, p-p65, or PARP in nuclear lysates were measured by Western blot assay with the indicated antibodies. (**B**) The indicated protein levels in 293T cells or CSAD KO cells. (**C**) The indicated protein levels in CSAD overexpressing cells or control cells. (**D**) Bone marrow-derived macrophages (BMDMs) from CSAD KO or B6 mice were differentiated and infected with PR8 (MOI = 1). Cells were lysed at indicated time points, and the levels of the indicated protein in total cell lysates or nuclear lysates were measured by Western blot assay. (**E**) A549 cells were transfected with control siRNAs or CSAD siRNAs, the indicated cells were harvested at 24 hpi, and the expression levels of CSAD were detected by Western blot assay. (blank: A549 cells with no treatment; lipo: A549 treated only with Lipofectamine 2000; NC: A549 cells were transfected with negative control siRNA provided by the company. (**F**) Changes in protein levels in CSAD knockdown A549 cells after PR8 infection. A549 cells were transfected with CSAD siRNAs. Twenty-four hours post-transfection, cells were infected with PR8 at MOI = 1. The levels of IKKα, IKKβ, p-IKKα, p-IKKα/β, IκB-α, p-IκB-α, CSAD, and β-actin in total cell lysates, and p65, p-p65, or PARP in nuclear lysates were measured by Western blot assay with the indicated antibodies. (**G**) CSAD KO 293T cells were transfected with pCDNA-flag-CSAD. Twenty-four hours post-transfection, cells were infected with PR8 at MOI = 1. The levels of IKKα, IKKβ, p-IKKα, p-IKKα/β, IκB-α, p-IκB-α CSAD, and β-actin in total cell lysates and p65, p-p65, or PARP in nuclear lysates were measured by Western blot assay with the indicated antibodies. The data correspond to one representative experiment from at least three independent experiments. (**H**) CSAD overexpressing cells or control 293T cells were infected with PR8 (MOI = 1). The culture supernatants were collected at 6 and 12 hpi. Levels of IL-6 in supernatants were measured by ELISA kit according to the manufacturer’s instructions. (**I**) Levels of IL-6 in the BMDM culture supernatant after PR8 infection (MOI = 1) at the indicated time points were measured by the ELISA kit. (**J**) 293T cells or CSAD KO 293T cells were infected with PR8, the supernatants were collected at 24 hpi, and the virus titers in the supernatants were measured by plaque assay. (**K**) Control A549 cells or CSAD knockdown A549 cells were infected with PR8, the supernatants were collected at 24 hpi, and the virus titers in the supernatants were measured by plaque assay. Data are representative of at least three independent experiments, and data points indicate means ± SEM. *P < 0.05, **P < 0.01; ***, *P* < 0.001.

To examine the detailed mechanisms by which CSAD intervenes in the NF-κB signaling pathway after infection, we generated CSAD KO 293T cells by the CRISPR/Cas9 system. A monoclonal KO cell line was obtained with a 128 bp deletion at exon 4 of the CSAD gene (Gene ID: 51380) allele, leading to early termination of CSAD expression. Results of agarose gel electrophoresis, and Western blot of 293T CSAD KO cells showed that the CSAD KO cell line was successfully constructed (Fig. 4A).

IKKα/β/γ complexes are important in the NF-κB signaling pathway as they can phosphorylate the downstream IκBα and then trigger translocation of p-p65 into the nucleus to regulate gene expression. This process depends mainly on IKKα/β phosphorylation in the canonical NF-κB pathway. As CSAD interacted with IKKα but not IKKβ (Fig. 3), we detected the change in main adapter proteins in the NF-κB signaling pathway. CSAD KO 293T cell line or WT 293T cells were infected with PR8 at MOI = 1, and whole cell lysis supernatants or nuclear lysis supernatants were harvested at different time points. Phosphorylation of IKKα and IKKα/β was detected in both cells after 3 hpi and increased with time. However, phosphorylation of IKKα and IKKα/β was enhanced in CSAD KO cells, especially at early time points, compared with that of WT cells. Consistently, phosphorylation of IκBα also increased in KO cells. Furthermore, downstream p65 phosphorylation and nuclear translocation enhanced in the KO cells as well. However, the activation of the noncanonical pathway p52 had no significant differences between both cells, indicating that CSAD might mainly affect the canonical NF-κB pathway (Fig. 4B; Fig. S3A). The data indicated that in the absence of CSAD, phosphorylation of IKKα was enhanced and the stimulation of the downstream signaling pathway was promoted.

Next, 293T cells were transiently transfected with pCDNA-CSAD-Flag to overexpress CSAD and then infected with PR8. Phosphorylation of IKKα and IKKα/β and the downstream phosphorylation of IκBα and subsequently p-p65 translocated into nuclear were all inhibited in CSAD-overexpressing cells compared with that of WT cells. Meanwhile, the activation of the noncanonical pathway p52 had no significant differences between both cells (Fig. 4C; Fig. S3B). Therefore, overexpression of CSAD inhibited the phosphorylation of IKKα and reduced the stimulation of the downstream signaling pathway. To further verify whether this characteristic is consistent in different types of cells, we obtained bone marrow-derived macrophage (BMDM) from CSAD KO mice or B6 mice and then infected those cells with PR8. As shown in Fig. 4D and Fig. S3C, phosphorylation of IKKα and IKKα/β increased at 3 hpi in CSAD KO BMDM, compared with that of WT BMDM. Consistently, phosphorylation of IκBα also increased, and phosphorylated p65 into the nuclear region also enhanced in CSAD KO BMDM. Again, there were no significant differences between p52 in both cells. Thus, after PR8 infection, the activation of IKKα, IκBα, and p65 was promoted in CSAD KO BMDM.

To further verify that CSAD limits the phosphorylation of IKKα and then the downstream signaling pathway, we knocked down CSAD expression in A549 cells by siRNA. CSAD expression was reduced in A549 cells at 24 hours post-transfection (Fig. 4E). Then, the cells were infected with PR8 at MOI = 1. As shown in Fig. 4F and Fig. S4A, phosphorylation of IKKα and IKKα/β increased in CSAD knockdown cells compared with that of control A549 cells. Consistently, phosphorylation of IκBα increased, and nuclear phosphorylated p65 also enhanced in the CSAD knockdown cells. Furthermore, we rescued the expression of CSAD in 293T CSAD KO cells, and the expression of CSAD inhibited the early phosphorylation of IKKα and then of the downstream signaling pathway (Fig. 4G; Fig. S4B).

Our previous results demonstrated that CSAD, rather than taurine, plays a crucial role in the phenotype of the KO mice after IAV infection. We further determined whether taurine affects the signaling pathway *in vitro*. Cells were treated with taurine at the indicated concentrations and then infected with PR8 at MOI = 1. Main adapter proteins were detected by Western blot at 6 hpi. As shown in Fig. S5, taurine did not affect the signaling pathway.

The NF-κB signaling pathway modulates the expression of cytokines such as IL-6 after PR8 infection; therefore, we also measured the IL-6 level in culture supernatants. 293T cells were transfected with pCDNA-Flag-CSAD or control plasmid for 24 hours. Then, the cells were infected with PR8. Cell culture supernatants were collected at different time points. We found that the expression of IL-6 was increased after virus infection. However, IL-6 was significantly inhibited in CSAD overexpressing cells compared with that of control 293T cells at 6 and 12 hpi (Fig. 4H). Next, we detected IL-6 levels in the BMDM culture supernatant after PR8 infection at different time points. CSAD KO BMDM and WT BMDM were infected with PR8, and the supernatants were collected at different time points, and the concentrations of IL-6 were measured by ELISA. IL-6 levels were significantly enhanced in CSAD KO BMDM at 6, 12, and 24 hpi (Fig. 4I). We further detected virus titers. 293T cells or CSAD KO 293T cells were infected with PR8, the supernatants were collected at 24 hpi, and the virus titers in the supernatants were measured by plaque assay. As shown in Fig. 4J, the virus titers were significantly reduced in the CSAD KO 293T cells compared to those of 293T cells. In addition, the virus titers were also significantly reduced in A549 cells with CSAD knockdown (Fig. 4K). Thus, CSAD KO or knockdown repressed virus replication, which is consistent with the higher activation of the NF-κB signaling pathway. Collectively, these data showed that CSAD limits the NF-κB signaling pathway through IKKα during PR8 infection.

### CSAD interacts with IKKΑ at the kinase domain to limit the NF-κB signaling pathway.

**Fig 5:**
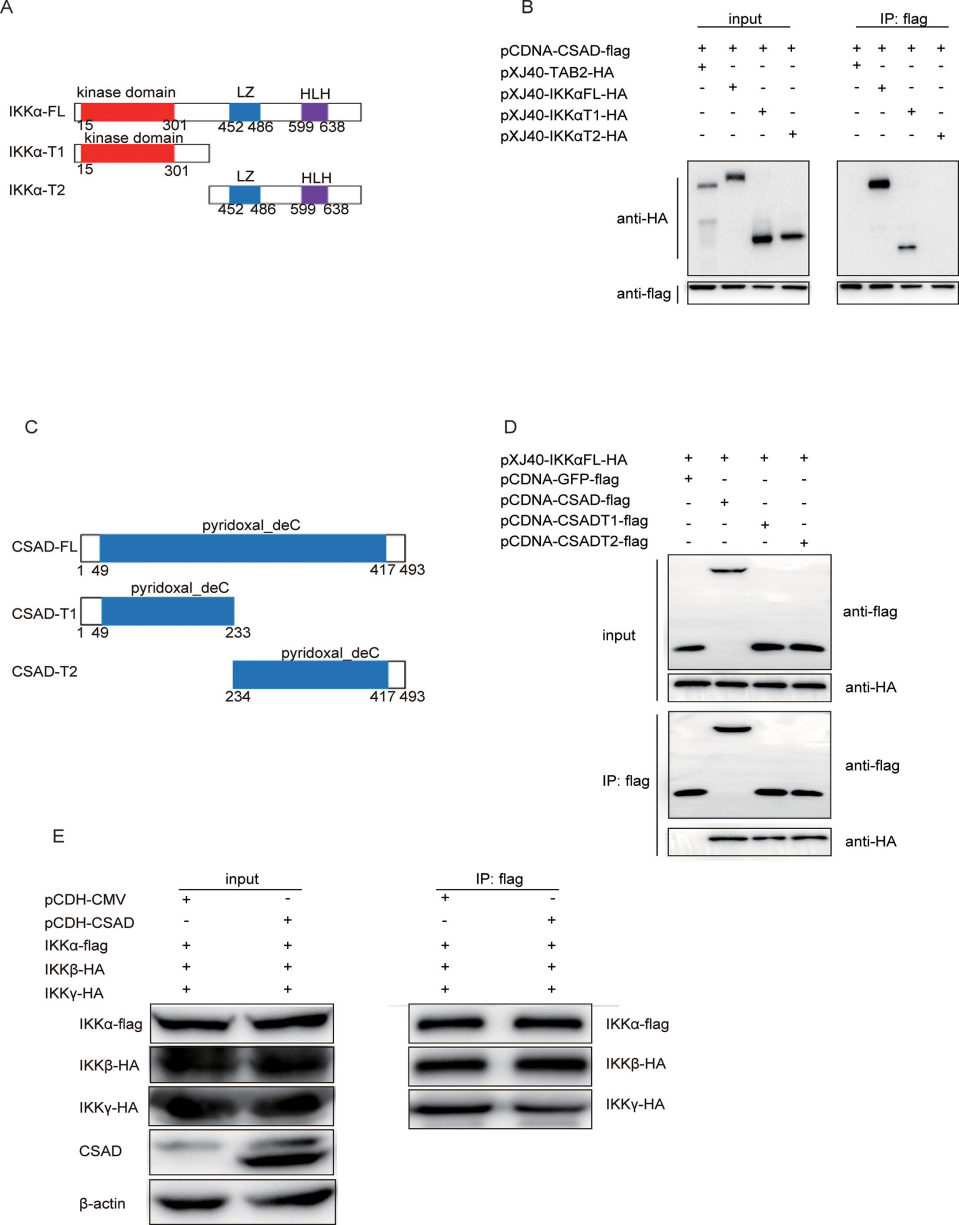
CSAD interacts with IKKα at its kinase domain. (**A**) Construction of IKKα truncations. Full-length IKKα was truncated at position 371 and got two truncations: IKKα-T1 (1–371 aa) and IKKα-T2 (372–745 aa). (**B**) Coimmunoprecipitation between CSAD and IKKα (FL, T1, and T2). 293T cells were transfected with pCDNA-CSAD-Flag, pXJ40-HA-IKKα FL/T1/T2. The cell lysates were incubated with anti-flag affinity gel and analyzed by Western blot with the indicated antibodies. (**C**) Construction of CSAD truncation. Full-length CSAD was truncated at position 233 to generate two truncations CSAD-T1 and CSAD-T2. (**D**) Coimmunoprecipitation between CSAD/T1/T2 and IKKα. 293T cells were transfected with pCDNA-CSAD-Flag/T1/T2 or with pXJ40-HA-IKKα. The cell lysates were incubated with anti-flag affinity gel and analyzed by Western blot with the indicated antibodies after immunoprecipitation. (**E**) Coimmunoprecipitation between CSAD and IKKα/β/γ. pCDH-CSAD was cotransfected with IKKα-Flag, IKKβ-HA, and IKKγ-HA in 293T cells, and the cell lysates were incubated with anti-flag affinity gel and analyzed by Western blot with the indicated antibodies after immunoprecipitation. Data are representative of at least five independent experiments.

IKKα is composed of three domains: the kinase domain, which is essential to phosphorylation downstream of the signaling pathway; the Leucine Zipper (LZ) domain; and the Helix-Loop-Helix (HLH) domain (Fig. 5A). As the results shown in Fig. 4B through G, phosphorylation of IKKα was enhanced in the absence of CSAD. To further investigate which domain of IKKα interacts with CSAD, we truncated IKKα at amino acid position 371 and got two truncations of IKKα: IKKα-T1 (1–371 aa), which includes the kinase domain, and IKKα-T2 (372–745 aa), which include the LZ and HLH domain (Fig. 5A). Phosphorylation of IKKα is related to the kinase domain from amino acid position 15 to 301, which contains two important serine sites that can be phosphorylated: S176 and S180. Coimmunoprecipitation was used to detect the interaction between CSAD and IKKα truncations. 293T cells were cotransfected with pCDNA-CSAD-Flag and HA-IKKα T1/T2 or -TAB2, which was used as a control vector. As shown in Fig. 5B, CSAD interacted with IKKα full length (CSAD-FL) and IKKα-T1, but not IKKα-T2, demonstrating that CSAD interacts with the IKKα kinase domain. On the other hand, since CSAD just has a catalytic activity domain, truncated CSAD was also constructed at the position of 233 (Fig. 5C) to find which CSAD domain interacts with IKKα, and these CSAD truncations were also used in coimmunoprecipitation. CSAD-T1 and T2 both interacted with IKKα *in vitro* (Fig. 5D), indicating both CSAD-T1 and T2 fragments are required for efficient CSAD-IKKα association.

Because the IKK complex plays an important role in the stimulation of the downstream pathway, we then further determined whether CSAD might affect the interaction between IKKα/β/γ. We cotransfected pCDH-CSAD and IKKα-Flag, or HA-IKKβ or -IKKγ, and used coimmunoprecipitation to detect whether CSAD might influence the formation of the IKK complex. We found that IKKγ slightly decreased with CSAD overexpression (Fig. 5E), indicating that CSAD may affect the formation of the IKK complex to a very low extent. In summary, these results demonstrated that CSAD suppresses the downstream NF-κB signaling pathway, mainly due to the interaction with IKKα at its kinase domain.

### CSAD is a general inhibitor of excessive inflammation.

**Fig 6:**
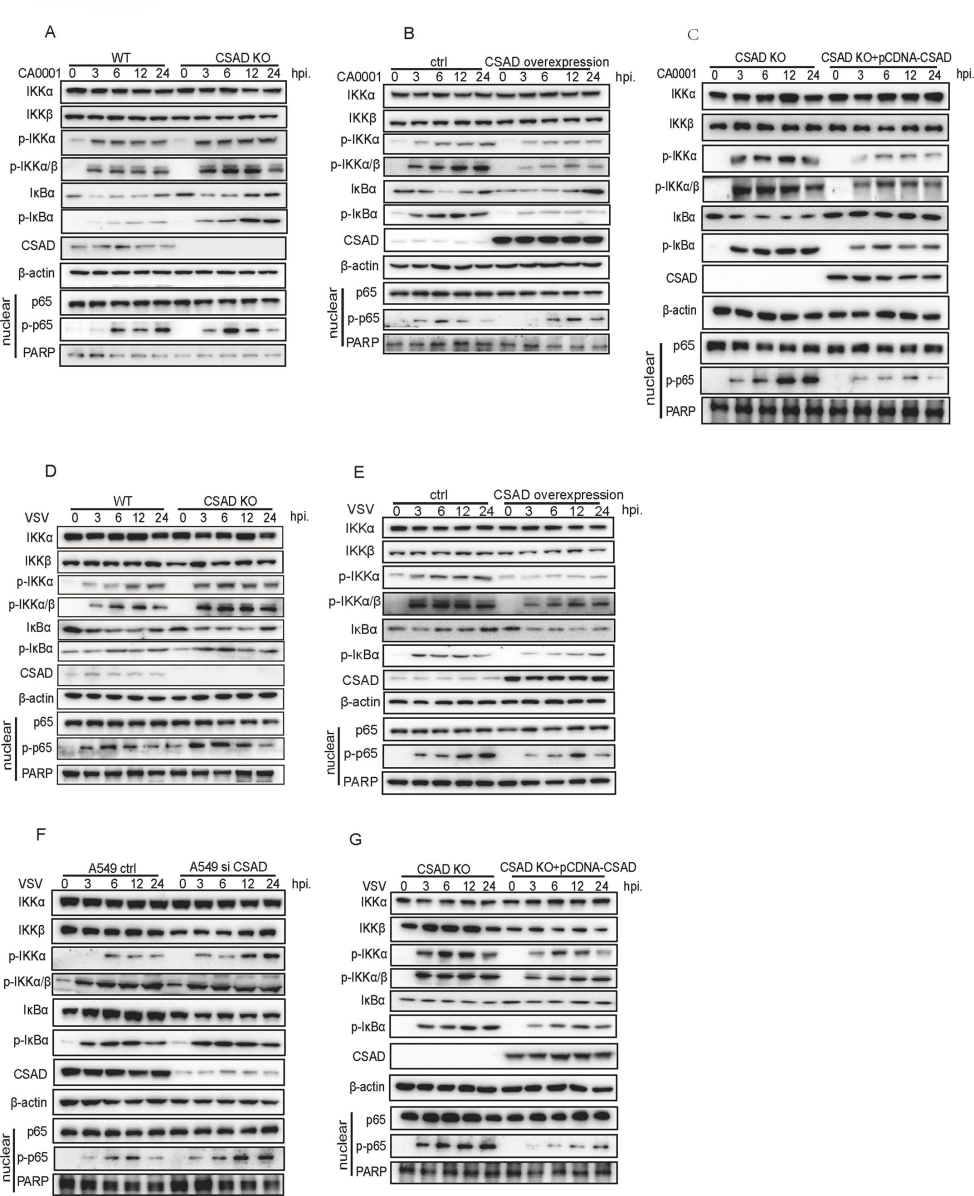
CSAD limits the fast and strong activation of the NF-κB signaling pathway in cells after viral infections. (**A–C**) 293T cells, CSAD KO cells, CSAD overexpressing cells, or CSAD KO 293T cells transfected with pcDNA-flag-CSAD were infected with CA0001 at MOI = 1, respectively. Cells were lysed at the indicated time points, and the levels of IKKα, IKKβ, p-IKKα, p-IKKα/β, IκB-α, p-IκB-α, CSAD, or β-actin in total cell lysates; p65, p-p65, or PARP in nuclear lysates were measured by Western blot assay with the indicated antibodies. (**A**) The indicated protein levels in 293T cells or CSAD KO cells. (**B**) The indicated protein levels in CSAD overexpressing cells or control cells. (**C**) The indicated protein levels in CSAD KO cells or CSAD KO cells transfected with pcDNA-flag-CSAD to rescue the expression of CSAD. (**D–G**) 293T cells, CSAD KO cells, CSAD overexpressing cells, CSAD KO 293T cells transfected with pcDNA-flag-CSAD, and A549 cells or A549 cells transfected with CSAD siRNAs were infected with VSV at MOI = 1, respectively. Cells were lysed at the indicated time points, and the levels of IKKα, IKKβ, p-IKKα, p-IKKα/β, IκB-α, p-IκB-α, CSAD, or β-actin in total cell lysates; p65, p-p65, or PARP in nuclear lysates were measured by Western blot assay with the indicated antibodies. (**D**) The indicated protein levels in 293T cells or CSAD KO cells. (**E**) The indicated protein levels in CSAD overexpressing cells or control cells. (**F**) The indicated protein levels in control A549 cells or A549 cells transfected with CSAD siRNAs. (**G**) The indicated protein levels in CSAD KO cells or CSAD KO cells transfected with pcDNA-flag-CSAD to rescue the expression of CSAD. Data are representative of at least five independent experiments.

According to our previous results, CSAD inhibits excessive inflammatory responses by interacting with IKKα after PR8 infection. We further investigated whether CSAD functions as a broad-spectrum inhibitor to restrain NF-κB signaling during other virus infections or stimulations. First, we infected CSAD KO cells with A/Changchun/01/2009(H1N1, CA0001), which is another strain of IAV. We found that in CSAD KO cells, phosphorylation of IKKα and IKKα/β was enhanced, phosphorylation of downstream IκBα was also increased, and translocation of p-p65 into the nuclear region enhanced (Fig. 6A; Fig. S6A). In contrast, when CA0001 was used to infect CSAD overexpressing 293T cells, which were transiently transfected by pCDNA-Flag/-CSAD-Flag, phosphorylation of IKKα and IKKα/β was inhibited, phosphorylation of downstream IκBα decreased, and subsequent translocation of p-p65 into the nuclear region was also restrained (Fig. 6B; Fig. S6B). Again, the restored expression of CSAD into CSAD KO 293T cells suppressed early activation of IKKα and downstream proteins after CA0001 infection (Fig. 6C; Fig. S6B).

Second, we infected CSAD KO 293T cells with vesicular stomatitis virus (VSV). We found that in CSAD KO cells, phosphorylation of IKKα and IKKα/β enhanced, and phosphorylation of downstream IκBα also increased, and subsequent translocation of p-p65 into nuclear enhanced (Fig. 6D; Fig. S7A). Contrarily, in CSAD overexpressing cells, phosphorylation of IKKα and IKKα/β was inhibited, phosphorylation of downstream IκBα decreased, and p-p65 in nuclear was suppressed after VSV infection (Fig. 6E; Fig. S7B). We further used siRNA to knock down CSAD expression in A549 cells. The results were similar to that of CSAD KO 293T cells after VSV infection (Fig. 6F; Fig. S7C). Moreover, the restored expression of CSAD in CSAD KO 293T cells suppressed the early activation of IKKα and downstream adapter proteins after VSV infection (Fig. 6G; Fig. S7D).

**Fig 7:**
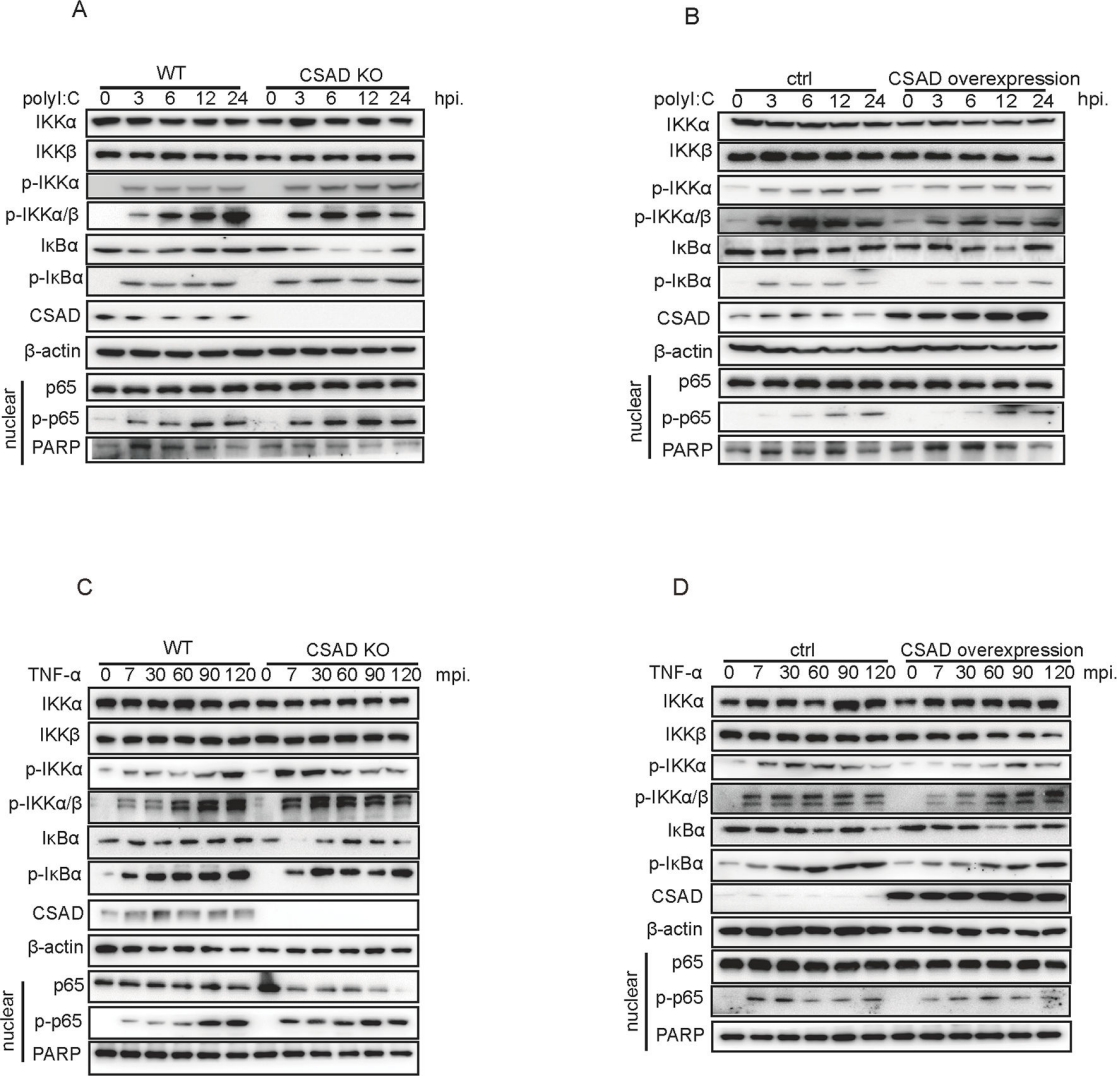
CSAD limits the fast and strong activation of the NF-κB signaling pathway in cells after stimulations. (**A and B**) The indicated cells were transfected with polyI:C. Cells were lysed at the indicated time points, and levels of signaling pathway protein were measured by Western blot assay. (**A**) The indicated protein levels in 293T cells or CSAD KO cells. (**B**) The indicated protein levels in CSAD overexpressing cells or control cells. (**C and D**) The indicated cells were treated with TNF-α (10 ng/mL). The levels of the indicated proteins were measured at the indicated time points by Western blot assay. (**C**) The indicated protein levels in CSAD KO or 293T cells. (**D**) The indicated protein levels in CSAD overexpressing cells or control cells. Data are representative of at least five independent experiments.

Third, polyI:C was used to imitate RNA virus infection to detect whether CSAD affects the NF-κB signaling pathway. PolyI:C was transfected into 293 T WT/CSAD KO cells. The phosphorylation of IKKα and IKKα/β was enhanced in CSAD KO cells at early time points. Phosphorylation of downstream IκBα also increased, and translocation of p-p65 into the nuclear enhanced in KO cells (Fig. 7A; Fig. S8A). Next, we cotransfected polyI:C and pCDNA-Flag/Flag-CSAD into 293T cells. As shown in Fig. 7B and Fig. S8B, phosphorylation of IKKα and downstream protein activations was suppressed in CSAD overexpressing cells.

Finally, we further investigated whether CSAD engages in the NF-κB signaling pathway after cytokine stimulation. Since cytokines can directly bind to cell surface receptors to trigger the activation of the signaling pathway, it takes a shorter time than viral infection to activate NF-κB signaling. Previous studies showed that in MEFs, IKK activity was detectable at 5 minutes after TNF-a stimulation (45). CSAD KO 293T cells or WT 293T cells were stimulated with TNF-α, whole-cell lysis supernatant, and nuclear lysis supernatant were collected at different time points. We found that phosphorylation of IKKα and IKKα/β was enhanced at 7 minutes post-stimulation (mps) after cytokine stimulation, and then the phosphorylation of downstream IκBα and phosphorylated p65 in nuclear was also increased in CSAD KO cells (Fig. 7C; Fig. S8C). Next, 293T cells were transiently transfected with pcDNA-CSAD-Flag and then stimulated with TNF-α. The phosphorylation of IKKα and IKKα/β and downstream signaling were inhibited in CSAD-overexpressing cells (Fig. 7D; Fig. S8D). Collectively, these data indicated that with different stimulations, CSAD generally suppresses the NF-κB signaling pathway by restricting the early phosphorylation of IKKα.

**Fig 8:**
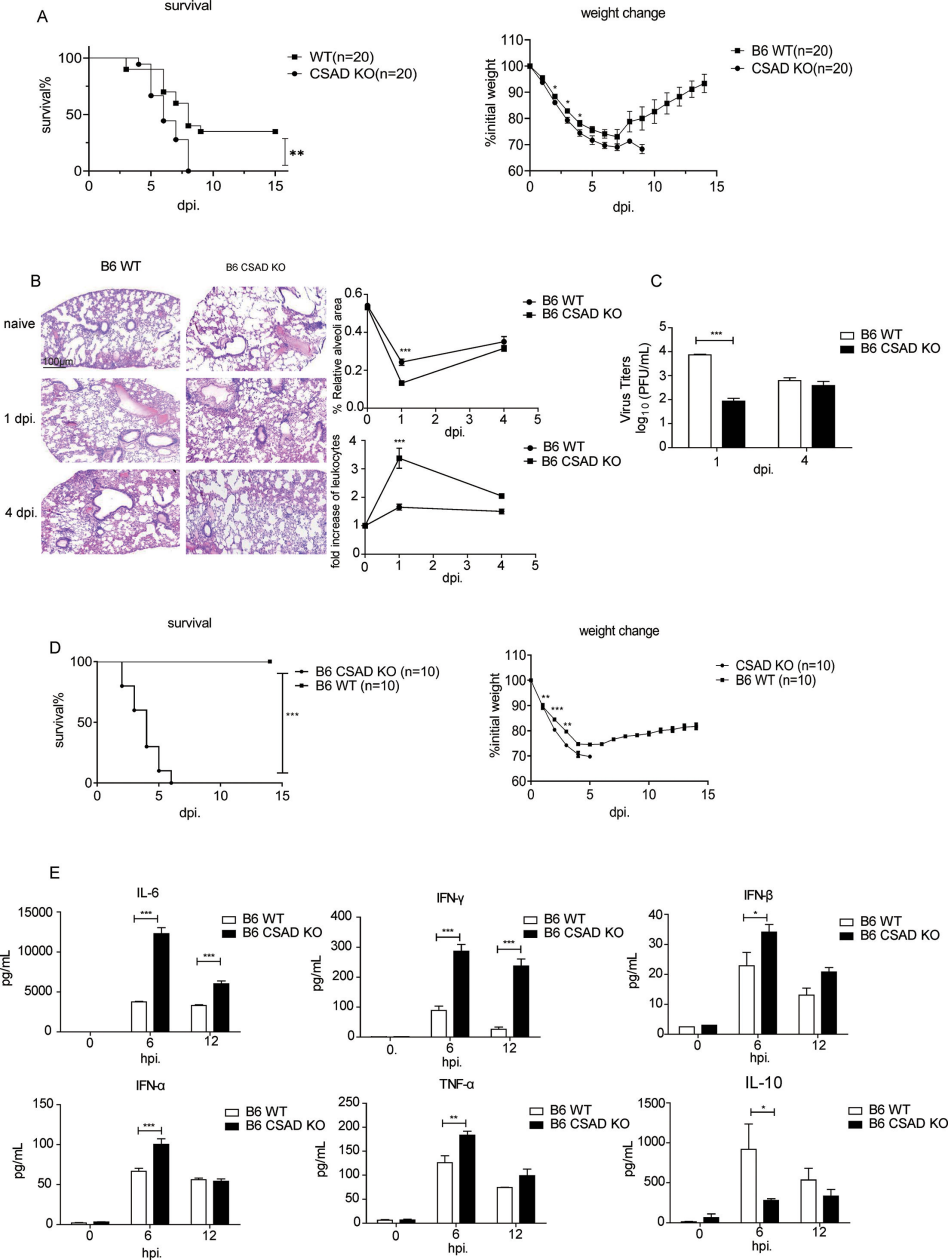
CSAD KO mice are more vulnerable to VSV infection and LPS stimulation. (**A–C**) CSAD KO mice and B6 mice were infected with 1 × 10^7^ pfu VSV. (**A**) Weight loss and survival rate of CSAD KO and B6 mice. Data are from three independent experiments, with 5–9 mice per group in each experiment. *, *P* < 0.05; **, *P* < 0.01; ***, *P* < 0.001. (**B**) Hematoxylin-eosin (H&E) staining of the lung sections at 1 and 4 dpi (original magnification ×10; scale bars, 100 µm) from CSAD KO mice or B6 mice (left), and statistics analysis of relative alveoli area and fold change of leukocyte infiltrate (right). (**C**) Viral titers of pulmonary homogenates from CSAD KO mice or B6 mice. (**D and E**) CSAD KO mice and B6 mice were stimulated with LPS (30 mg/kg) by intraperitoneal injection. (**D**) Weight loss and survival rate of CSAD KO mice and B6 mice. Data are from two independent experiments, with five mice per group in each experiment. *, *P* < 0.05; **, *P* < 0.01; ***, *P* < 0.001. (**E**) Cytokine levels in the sera of CSAD KO mice or B6 mice. CSAD KO mice or B6 mice were injected with LPS. Blood was collected at the indicated time points, and sera were prepared to measure the levels of IFN-γ, IFN-α, IFN-β, IL-6, TNF-,α and IL-10. Data are presented as the mean ± SEM from three independent experiments. *, *P* < 0.05; **, *P* < 0.01; ***, *P* < 0.001.

Next, we further investigated this general function of CSAD *in vivo*. First, we infected CSAD KO mice and WT mice with VSV at 1 × 10^7^ pfu per mouse and detected the weight change and survival rate. We observed that CSAD KO mice were much more vulnerable to VSV infection compared with WT B6 mice as the weight loss of CSAD KO mice was much more severe, and all the mice died within 1 week (Fig. 8A). We next sacrificed the infected mice at 1 dpi and 4 dpi after VSV infection, and lung samples were prepared for H&E staining and virus loads. More severe inflammation was observed in CSAD KO mice compared with that of WT mice after VSV infection at 1 dpi (Fig. 8B, left panel). The relative alveoli area was reduced, and the fold change of infiltration of leukocytes was increased in CSAD KO mice (Fig. 8B, right panel). Consistent with the severe inflammatory responses in CSAD KO mice at 1 dpi, the viral loads in the lungs of KO mice were significantly lower than those of B6 mice (Fig. 8C). The results were similar to PR8 infection.

We further used the lipopolysaccharide (LPS) inoculation model to determine the role of CSAD in bacterial infection. CSAD KO and B6 mice were intraperitoneally (i.p.) injected with LPS, which is a component of the bacteria cell wall, at a dose of 30 mg/kg. We found that CSAD KO mice were much more vulnerable compared with B6 WT mice (Fig. 8D) as the weight loss in CSAD KO mice was much faster after LPS stimulation than in WT mice; all the CSAD KO mice died within 1 week. Moreover, we investigated the cytokine level in serum after LPS stimulation. CSAD KO/WT mice were bled, and sera were collected at the indicated time points (6 h and 12 h) after the LPS injection. Inflammatory cytokines were measured by the Legendplex kit. The levels of IL-6 and IFN-γ in CSAD KO mice were higher than those in WT mice at 6 hps and 12 hps, and type I interferon (IFN-α and β) in CSAD KO mice was much higher at 6 hps but not at 12 hps. TNF-α level was also higher in CSAD KO mice at 6 hps. However, IL-10, an anti-inflammatory cytokine, was higher in WT mice than that in CSAD KO mice at 6 hps (Fig. 8E). These results indicated that inflammatory cytokines were highly produced after LPS stimulation in the absence of CSAD. Thus, all these data demonstrated that CSAD limits early strong NF-κB signaling activation in various pathogen infections and stimulations, and it is a novel general inhibitor for excessive inflammatory responses during a broad range of virus infections and stimulations.

## DISCUSSION

Viral and bacterial infections have caused high morbidity and mortality worldwide (1). Innate immunity is the first line of defense against infections. It can not only restrain the early replication and transmission of pathogens but also stimulate and influence the nature of the subsequent adaptive immune responses. The early local reaction of innate immunity is the inflammatory response. Inflammation produces a variety of systemic changes in the host that enhance the ability of the innate immune system to eradicate infection. The speed and magnitude of the inflammatory responses are essential for pathogen clearance and disease recovery. However, dysregulated excess systemic cytokine production is harmful and results in syndromes such as cytokine storm (7), sepsis (46), or tissue damage, which may even cause the death of the host. This has been found in many infectious diseases; for example, in COVID-19 patients, an excessive induction of proinflammatory cytokines, such as interferon or interleukin, has been detected after infection, and acute respiratory distress syndrome (ARDS) has been found in many COVID-19 cases (47). Hence, it is important to prevent excessive inflammatory responses and balance pathogen clearance and immunopathology.

The capacity to regulate inflammatory responses varies between different hosts. Several studies have demonstrated that during IAV infection, the early innate immune responses vary in different mouse strains (27). 129 mice exhibit stronger immune responses after viral infection, which causes excessive inflammation with the secretion of cytokines including IL-6 and IFN-α, resulting in more severe lung injury and higher mortality compared with B6 mice (28). Furthermore, it has been reported that host factors are important in virus replication during infection by genome-wide RNAi screening (29). For the same pathogenic microorganism infection, some hosts initiate a moderate immune response to control the infection and recover, while some hosts might generate excessive immune responses, leading to severe tissue injury or even death. The reasons for these differences might be very complex and multifactorial, and we still know very little about them. However, host-differentiated regulatory factors might affect the initial strength of inflammatory responses.

CSAD is involved in the second step of the taurine synthesis pathway, and so far, research about CSAD has concentrated mainly on its structural information, kinetic properties, and metabolic functions (33). Whether CSAD plays any role in the antiviral process has not been investigated. In this study, we generated CSAD KO mice in the B6 background and maintained the health of the KO mice by feeding them taurine. Strikingly, we found that knockout CSAD in B6 mice results in faster and stronger inflammatory responses after IAV infection, including severe lung injury, lower virus titers, and higher cytokine concentration at an earlier time point post-infection, a phenotype similar to that of IAV-susceptible 129 mice. We further demonstrated that the absence of CSAD, rather than taurine, plays a crucial role in the phenotype of the KO mice after viral infection. Interestingly, the expression level of CSAD is much higher in B6 mice than that of 129 mice. These data suggest that host differentially expressed factor CSAD may be involved in regulating the intensity of inflammatory responses.

Activation of the NF-κB signaling pathway is crucial to inflammatory responses. Many factors are involved in the regulation of the pathway and constitute elaborate regulatory networks, such as regulating of IκB proteins, IKK complex, and post-translational modifications (48). We found that CSAD limits the early and stronger activation of NF-κB signaling after stimulation by directly interacting with IKKα. CSAD interacts with IKKα at the kinase domain to inhibit its fast and strong phosphorylation and downstream activation of the NF-κB signaling pathway. In CSAD KO mice, the transcripts of TNF-α, IL-1β, and IL-6 are significantly higher than those of the B6 mice after PR8 infection. In addition, secretions of IL-6 and TNF-α are markedly higher in the CSAD KO mice than those of the B6 mice after LPS stimulation. IKKβ has long been viewed as the dominant IKK in the canonical NF-κB signaling pathway (48, 49). However, recent studies have shown that IKKα can also play a major role in canonical NF-κB signaling (50, 51). IKK-α has been shown to affect canonical NF-κB signaling by directly phosphorylating histone H3. This process enhances the transcription of a variety of genes, including IL6, and is independent of IKK-β (52, 53). Additionally, IKK-α has been shown to directly phosphorylate the NF-κB subunit p65 at Ser536 (54). We found that CSAD-IKKα interaction limits early phosphorylation of IKKα and IKKα/β, and downstream canonical NF-κB, as the activation of noncanonical pathway p52 is not affected by CSAD. As we did not get IKKβ-specific phosphorylation Ab, it was hard to distinguish whether the phosphorylation of IKKβ is also enhanced in the absence of CSAD. The detailed mechanisms of IKK-α inhibited by CSAD deserve further investigation. Nonetheless, our data have shown that CSAD directly interacts with IKKα to limit its early phosphorylation.

Strikingly, type I interferon (IFN-I) production is also affected by CSAD. In CSAD KO mice, the mRNA level or protein level of IFN-I is also marked higher than that of B6 mice after virus infection or LPS stimulation, respectively. Previous studies have shown that IKKα plays a critical role in TLR7-/9-induced IFN-I production, but not in the production of proinflammatory cytokines, indicating IKKα is a unique molecule involved in TLR7-/9-MyD88-dependent type I IFN production through DC subset-specific mechanisms (55). The increased level of IFN-I in the CSAD KO mice after virus infection is consistent with the previous studies showing IKKα plays a critical role in IFN-I production, while the increased proinflammatory cytokines in the CSAD KO mice are not consistent with the previous finding (55). The different experiment settings may play a role in the differences. Meanwhile, all these data indicate that IKKα plays an important role in innate signaling pathways.

Importantly, CSAD is highly conserved in mammals. For example, humans and mice share 88.26% homology in cDNA; humans and pigs share 89.07% homology in cDNA. In our studies, we found that the basal expression levels of CSAD vary in different mouse strains. We still do not know the level of CSAD in individual human beings and its correlation to the outcome of pathogen infection. Because CSAD interacts with IKKα and limits its fast and strong phosphorylation and downstream activation of the NF-κB signaling pathway, it may fine-tune the strength of NF-κB signaling in human beings as well. Besides the differences in gene expression, gene variants have also been associated with the risk of certain diseases. For example, a single-nucleotide polymorphism (SNP) of IFITM3 (rs12252-C) has been associated with the risk of severe influenza (30). Recently, two common IFITM3 polymorphisms (rs34481144 C/T and rs12252 A/G) are associated with different risks for hospitalization after SARS-CoV-2 infection (56). By three genome-wide association meta-analyses, 13 genome-wide significant loci have been reported to be associated with SARS-CoV-2 infection and severe manifestations of COVID-19 (57). Previous analysis revealed that the minor A allele of rs3782151 in CSAD might be associated with fulminant type 1 diabetes (40). Whether the polymorphisms of CSAD might have associations with the outcome of infectious diseases deserves further study.

**Fig 9:**
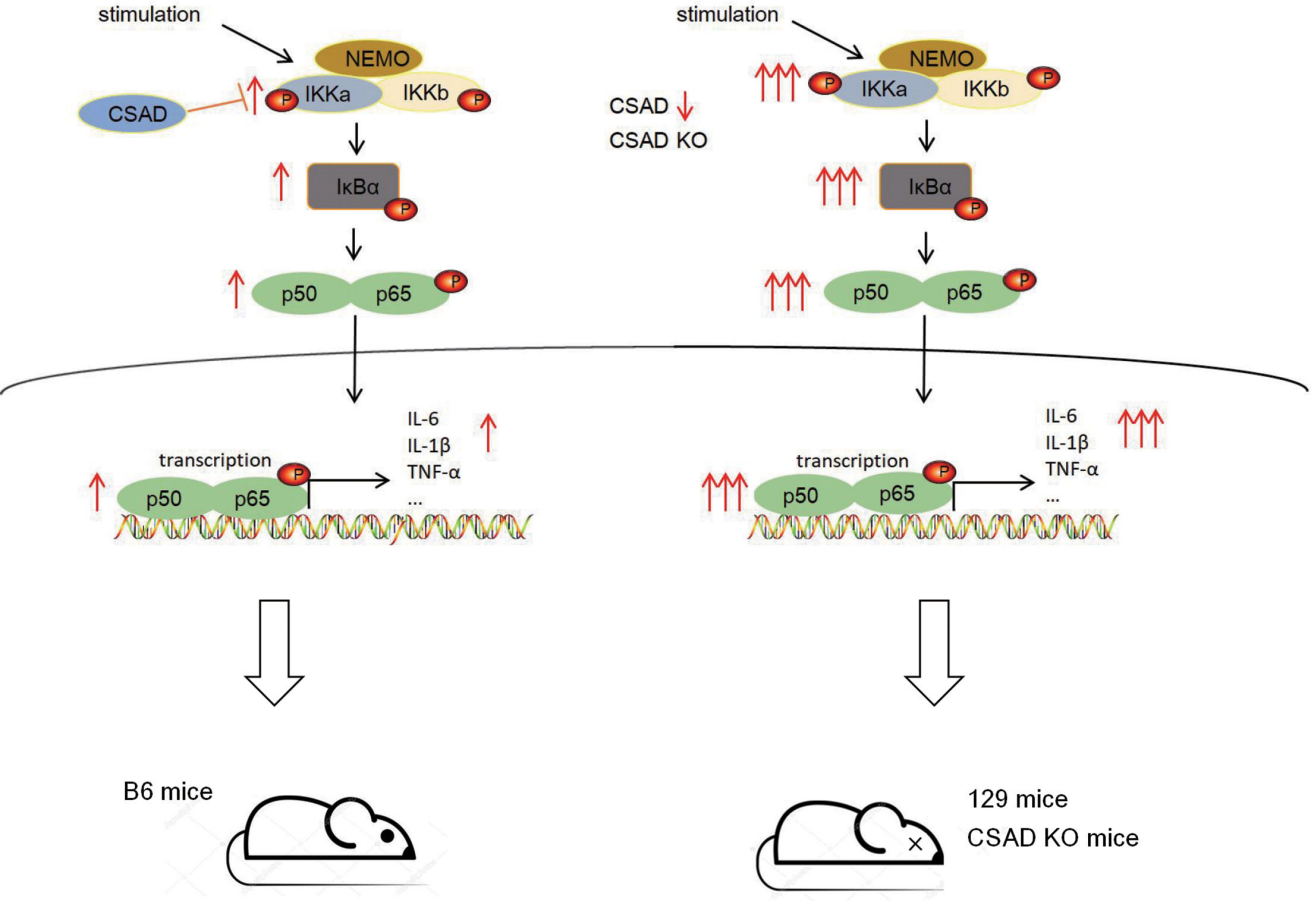
The mechanisms of CSAD regulate the strength of the NF-κB signaling pathway. The NF-κB signaling pathway can be stimulated by a series of stimuli, such as virus infection, and initiate the transcription of various genes that play important roles in inflammatory responses. In B6 mice, the CSAD protein level is higher, and CSAD interacts with the IKKα kinase domain and limits the degree of its phosphorylation and downstream activation of the NF-κB signaling pathway, contributing to moderate inflammatory responses to control virus infection. In 129 mice or CSAD KO mice, the CSAD level is lower or aborted, which results in a higher degree of IKKα phosphorylation at early time and downstream activation of the NF-κB signaling pathway. The production of a large amount of inflammatory cytokines leads to excessive inflammation and pathological damage of tissues and organs after virus infection.

We show here that CSAD expression acts as a fine tuner for the activation of the NF-κB signaling pathway. CSAD interacts with the IKKα kinase domain and limits the degree of its phosphorylation and downstream activation of the NF-κB signaling pathway. CSAD protein level is higher in B6 mice, and the early inflammatory responses in B6 mice are moderate compared to that of 129 mice or CSAD KO mice after virus infection or LPS stimulation. In contrast, CSAD level is lower or aborted in 129 mice or CSAD KO mice, which results in a higher degree of IKKα phosphorylation and downstream activation of the NF-κB signaling pathway. The production of a large amount of inflammatory cytokines leads to excessive inflammation and pathological damage of tissues and organs after virus infection (Fig. 9). Our studies reveal an important role of CSAD in regulating innate immune responses, adding a novel regulator to the complex networks of the NF-κB signaling pathway. Furthermore, our results also help us further understand the variations in the innate immune responses among individuals and provide a new perspective for the development of new drugs or therapies for infectious diseases.

## MATERIALS AND METHODS

### Cells

293T cells (human embryonic kidney cell line, ATCC, CRL-3216), MDCK cells (Madin-Darby canine kidney cell line, ATCC, CCL-34), A549 cells (human airway epithelial cell line, ATCC, CCL-185), HeLa cells (human adenocarcinoma cell line, ATCC, CCL-2), and DF-1 cells (Chicken embryonic fibroblast cell line, kindly provided by Dr. Zhengfan Jiang, Peking University, PR China) were cultured in Dulbecco’s modified Eagle’s medium (DMEM) with 10% fetal bovine serum (Gibco, Paisley, United Kingdom), 2 mM L-glutamine (Hyclone, USA), 100 μm non-essential amino acids (Hyclone, USA), 10 mM HEPES (Sigma, Germany), 0.05 mM β-mercaptoethanol (Sigma, Germany), 100 IU/mL penicillin, and 100 μg/mL streptomycin (Invitrogen, USA). All cells were cultured at 37°C in 5% CO_2_.

### Virus and plaque assay

Influenza A/Puerto Rico/8/34 (H1N1, PR8) virus and A/Changchun/01/2009 (H1N1, CA00001) were propagated in chorioallantoic cavities of 10-day-old specific pathogen-free embryonated chicken eggs (Beijing Merial Vital Laboratory Animal Technology Co., Ltd) for 48–72 h at 37°C. Vesicular stomatitis virus (VSV) was kindly provided by Zhengfan Jiang (Peking University, Peking, PR China) and propagated as described previously (58). All the virus was stored at −80°C.

Virus titers were determined by MDCK (for PR8) and DF-1 cells (for VSV). The virus was incubated with indicated cells at 37°C for 1 hr and then removed by washing with PBS. Cell monolayers were overlaid with agar medium, which contains DMEM, 1 μg/mL TPCK (N-tosyl-L-phenylalanyl chloromethyl ketone)-treated trypsin and incubated at 37°C for 48 h. The plates were fixed with 4% paraformaldehyde for 30 min, and agar overlays were removed. Then, the plates were stained by staining buffer (0.1% crystal violet in 20% ethanol) for 15 min. Plaques were counted, and virus titers were calculated accordingly.

### Constructs and antibodies

Using published sequences, these vectors were constructed: CSAD gene (Gene ID: 51380) was cloned into pCDNA4.0-Flag, pCDH, and pGADT7, and its truncates (CSAD-T1 and CSAD-T2) were cloned into pCDNA4.0-Flag. IKKα was cloned into pXJ40-HA, pGBKT7, and pCDNA4.0-Flag, and its truncates (IKKα T1 and IKKα T2) were cloned into pXJ40-HA. IKKβ was cloned into pXJ40-HA and pGBKT7, and IKKγ was cloned into pXJ40-HA. GFP was cloned into pCDNA4.0-Flag. pGL3-luc-RIG I, pGL3-luc-NF-κB, pGL3-luc-IFN-β, and pGL3-luc-AP-1 reporter plasmids were constructed and stored in our lab. For pGL3-luc-NF-κB, the NF-κB-binding site was cloned into the pGL3-luciferase plasmid at the upstream of the luciferase gene. For the other reporter plasmids, the indicated promoter sequence or the binding site was cloned into the pGL3-luciferase plasmid at the upstream of the luciferase gene.

Goat Anti-HA-tag Polyclonal Antibody (A00168; IP application concentration 2–10 µg/mg of lysate) was purchased from GenScript Corporation. Mouse anti-Flag (M2; F3165, Application concentration 10 μg/mL) antibody was purchased from Sigma. Mouse anti-β-actin (C4; sc-47778, dilution range 1:100–1:1,000), anti-influenza A NP (sc-101352, dilution range 1:100–1:5000) antibodies, anti-IKKα (sc-52932, WB dilution range 1:100–1:1000; IP application concentration 1–2 μg/mL), anti-IkBa (sc-1643, dilution range 1:100–1:1,000), anti-p-p65 (sc-136548, dilution range 1:100–1:1,000), anti-p65 (sc-8008, dilution range 1:100–1:1,000) were from Santa Cruz Biotechnology. Mouse anti-CSAD (ab82613; dilution range 1:1,000–1:5,000), Rabbit anti-p-IKKα (ab138426, dilution range 1:500–1:1,000), anti-IKKα/β (ab194528, dilution range 1:500–1:1,000) were from abcam Inc. Rabbit anti-IKKβ (no. 8943, dilution range 1:1,000), anti-PARP (no. 9542, dilution range 1:1,000), anti-p100/p52 (no. 4882s, dilution range 1:1,000), and mouse anti-p-IkBa (no. 9246; dilution range 1:1,000) were from Cell Signaling Technology, Inc. Rabbit anti-M1 Ab was prepared as described previously (59). HRP-conjugated anti-mouse, anti-rabbit, and anti-goat IgG secondary antibodies, and Rabbit FITC-anti-IgG (123962, Antibody concentration 400 μg/mL, dilution range 1:2,500), Mouse TRITC-anti-IgG (125106, Antibody concentration 750 μg/mL, dilution range 1:200) were obtained from ZSGB-BIO (Beijing, China).

### Cell transfection and stimulation

293T cells were incubated at a 12-well plate and transfected with pCDNA4.0-Flag or pCDNA4.0-Flag-CSAD or siRNA (si1: GAGTCCAGATTACCACGAA; si2: GAAGGGTTTGAGCTAGTCA; si3: AGGACAAGTTCTACGATGT) with Lipofectamine 2000 (Invitrogen) according to the manufacturer’s instruction. Twenty-four hours later, cells were infected with PR8 (MOI=1), CA0001 (MOI=1), VSV (MOI=0.1), and TNF-α (10 ng/μL per well). Cells were collected at indicated time points (0, 3, 6, 12, and 24 hpi) and proceeded to subsequent experiments.

293T cells were co-transfected with pCDNA4.0-Flag or pCDNA4.0-CSAD-Flag and poly I:C with Lipofectamine 2000 according to the manufacturer’s instructions. Twenty-four hours later, cells were collected and proceeded to subsequent experiments.

To determine whether taurine affects NF-κB signaling *in vitro*, two groups of 293T cells were treated with different concentrations of taurine (0, 2, 4, 6, and 8 mM for each group), which were added into cell culture 6 days before infection. Then one group of cells was infected with PR8 at MOI=1 and was treated with taurine till 6 hpi. Then, cells were harvested and proceeded to subsequent experiments.

### Western blot assay

Cell lysis and isolation of total protein samples: The indicated cells were collected and washed with PBS, and an appropriate volume of cell lysis buffer (RIPA Lysis Buffer+1% Protease Inhibitor Cocktail+1% Phosphatase Inhibitor Cocktail; Beyotime-P0013B, CWBIO-CW2200S, CWBIO-CW2383S）was added. After the mixture was incubated on ice for 20 minutes, it was centrifuged at 12,000 rpm for 5 minutes at 4°C, and the supernatant was obtained as the total cellular protein.

Nuclear and cytoplasmic fractionation was performed following the manufacturer’s protocol (Beyotime, P0027), summarized as follows: Cells were collected and washed with PBS, incubated on ice for 10 minutes, vortexed for 5 seconds, and placed on ice for 1 minute. An appropriate volume of cytoplasmic extraction buffer B (containing 1% PMSF, ST506) was added, followed by vigorous vortexing for 5 seconds. The lysate was centrifuged at 12,000 rpm for 5 minutes at 4°C, and the resulting supernatant was collected as the cytoplasmic protein fraction. After completely aspirating the supernatant, the pellet was resuspended in an appropriate volume of nuclear protein extraction buffer (containing 1% PMSF), incubated on ice for 3 minutes, and vortexed for 30 seconds. This cycle of ice incubation and vortexing was repeated for a total of 30 minutes. Finally, the mixture was centrifuged at 12,000 rpm for 10 minutes at 4°C, and the supernatant was collected as the nuclear protein fraction.

The indicated protein lysates prepared as previously described were added into the SDS-PAGE loading buffer. The samples were boiled to 100°C for 10 min, and cell lysates were analyzed by 10% SDS-PAGE. Proteins were electrotransferred to polyvinylidene fluoride membranes and incubated with the first antibodies indicated in this article. Bound Abs were detected using an HRP-conjugated anti-mouse, anti-rabbit, or anti-goat IgG secondary Ab and ECL development (Pierce) according to the manufacturer’s protocol.

### Yeast two-hybrid assay

The yeast two-hybrid experiment was performed according to the matchmaker yeast two-hybrid system (Clontech) as previously described (60, 61). Briefly, IKKα and IKKβ were expressed as Gal4 DNA-BD fusion proteins in the pGBKT7 plasmid; CSAD was expressed as Gal4 AD fusion proteins in the pGADT7 vector. Bait- and prey-plasmids were transformed into Y2H yeast Gold strain. And then, bait- and prey-transformed strains (IKKα or IKKβ and CSAD) were mated, and the resulting diploids were selected in high stringency medium (SD/-Ade/-His/-Leu/-Trp/+AbA/+X-α-Gal, QDO/A/X). The interaction between p53 and SV40 large T-antigen (p53/T-antigen) was used as a positive control, and empty vectors were employed as negative controls. Positive protein-protein interactions result in blue colonies in the QDO/X/A plates.

### Coimmunoprecipitation

For interaction between CSAD and exogenous IKKα (full length, T1 and T2), we cotransfected with pCDNA4.0-CSAD-Flag, and pXJ40-IKKα-HA (full length, T1 or T2) in 293T cells with Lipofectamine 2000. After 36 h post-transfection, cells were lysed and centrifuged, and the supernatants were incubated with anti-flag M2 affinity beads (Sigma) for 3 h. The beads were washed four times with lysis buffer, and all samples were denatured by boiling, resolved by SDS-PAGE, and analyzed by Western blot assay with indicated antibodies. As for the interaction between truncated CSAD and IKKα, 293T cells were co-transfected with pXJ40-IKKα-HA and pCDNA4.0-CSAD-Flag (T1 and T2), after 36 h post-transfection, cells were treated as the procedure described above.

Furthermore, to investigate the interaction between CSAD and endogenous IKKα, we transfected pCDNA 4.0-CSAD-Flag in 293T cells. Cell lysates were treated with the same procedures described above. Also, for the influence of CSAD on the interaction between IKKα, IKKβ, and IKKγ, 293T cells were cotransfected with pCDH-CSAD, pCDNA4.0-IKKα-Flag, pXJ40- IKKβ-HA, and pXJ40- IKKγ-HA, and then cells were treated as the procedure described above.

### Mice and infection

CSAD knockout C57BL/6 (B6) mice were generated by Shanghai BIORAY LABORATORIES Inc, China. Genomic DNA from the transgenic mice tail tissues was used for genotyping by PCR. The primers used were as follows.

Forward: 5′-AGGGTCACGTCCTTTGTTTCC -3′;

Reverse: 5′-TAGTGTTGAGGCTCTCCGTGAT -3′

The protein expression level of CSAD in different tissues and cells was further verified by Western blot, as indicated. The mating pairs of CSAD KO mice were maintained by feeding with 0.05% taurine in their drinking water (41). B6 mice (6–8 weeks of age) were purchased from Vital River, China. All mice were housed in an animal facility under specific pathogen-free conditions. For infection experiments, mice were transferred to a biosafety level 2 room. Mice were infected as described previously (28). The weight loss and survival of infected mice were observed daily for 15 days post-infections. In addition to mice that were found dead, mice with a weight loss of > 30% were euthanized and recorded as dead.

To determine whether taurine plays a role in the phenotype of CSAD KO mice, a group of CSAD KO mice was continually treated with taurine daily by gavage administration (165 mg/kg) after weaning (44). A group of age-matched WT mice was daily treated with taurine 10 days before infection. Other groups of CSAD KO mice and WT mice did not receive taurine treatment. All the groups of mice were infected with 1×10⁶ pfu PR8. In the taurine groups, CSAD KO or WT mice were continually treated with taurine till 15 dpi. The weight change and survival rates were monitored daily after viral infection.

### Histopathology and immunohistochemistry

Indicated mice lung tissues were removed and fixed with 4% paraformaldehyde for at least 12 h, dehydrated in a series of graded alcohols, and embedded in paraffin. Tissue sections (8 μm) were cut and mounted on glass slides and stained as described previously (62) and then examined by microscopy Leica CS2 for histological changes. Relative alveoli area and fold change of infiltrated leukocytes in the lungs of infected mice were compared to that of the corresponding naive mice and were calculated and analyzed accordingly by ImageJ software.

### Immunofluorescence assay

To determine the localization of CSAD with IKKα or IKKβ, HeLa cells were transfected with those plasmids, as indicated. After 24 hours, the cells were washed three times with PBS, fixed in 4% paraformaldehyde for 10 minutes at room temperature, and permeabilized with 0.1% Triton X-100 for 5 minutes. After blocking in 1% BSA for 1 hour, the cells were incubated for 1 hour with mouse anti-flag and rabbit anti-myc antibody at room temperature. After washing with PBS, the cells were incubated for 1 hour with a TRITC/FITC-conjugated secondary antibody and then with DAPI (BioRoYee) for 5 min. The glass dishes were washed and mounted using a mounting medium (ab103746, Abcam). Images of the cells were observed with a Leica SP8 confocal microscope.

### ELISA

293T (WT or CSAD overexpressing) or BMDMs (B6 WT and B6 CSAD KO) were infected with PR8 at MOI=1, and supernatants were collected at the indicated time points. IL-6 levels were measured by the mouse IL-6 ELISA kit (Invitrogen) according to the manufacturer’s instructions.

### Quantitative real-time PCR

Mice were sacrificed, and total RNA was extracted from the lungs of infected mice with TRIzol reagent (Invitrogen). The first-strand cDNA was synthesized by using oligonucleotide primers, which were demonstrated in Table 1. Universal Probe Library probes were purchased from Roche. First-strand cDNA was synthesized using oligo-dT primers. qRT-PCR was performed using a LightCycler480 (Roche). The cycling conditions for real-time PCR were as follows: 95°C for 10 min, followed by 40 cycles of 95°C for 10 s, 60°C for 30 s, and 72°C for 1 s. The fold increase in mRNA expression was determined using the ΔΔCt method relative to the values for the naïve mice after normalization to GAPDH gene expression.

**TABLE 1:** Primers used in this study

Gene	Forward oligonucleotide	Reverse oligonucleotide	Probe
For mouse			
*IFN-α4*	TCAAGCCATCCTTGTGCTAA	GTCTTTTGATGTGAAGAGGTTCAA	3
*IFN-β*	CTGGCTTCCATCATGAACAA	AGAGGGCTGTGGTGGAGAA	18
*TNF-α*	TCTTCTCATTCCTGCTTGTGG	GGTCTGGGCCATAGAACTGA	49
*IL-1β*	AGTTGACGGACCCCAAAAG	AGCTGGATGCTCTCATCAGG	38
*IL-6*	GCTACCAAACTGGATATAATCAGGA	CCAGGTAGCTATGGTACTCCAGAA	6
*GAPDH*	TGTCCGTCGTGGATCTGAC	CCTGCTTCACCACCTTCTTG	80

### Cytokine and chemokine analysis

Mice were infected by PR8 or stimulated by LPS and bled at indicated time points. Cytokine and chemokine levels were measured using a Mouse Anti-virus Response Panel Mix (BioLegend) according to the manufacturer’s instructions.

### Statistical analysis

Statistical analysis was performed using Prism software (GraphPad). All statistical analyses were performed using an unpaired two-tailed Student’s *t* test or two-way ANOVA test as applicable. When applicable, data were displayed as mean ± SEM. *P* values < 0.05 were considered statistically significant.

## Data Availability

All relevant data are within the article.

## SUPPLEMENTAL MATERIAL

Fig. S1 jvi.00706-25-s0001.pdf Taurine did not affect CSAD KO mice phenotype after PR8 infection.

Fig. S2 jvi.00706-25-s0002.pdf CSAD is widely expressed in human and mouse tissues and organs.

Fig. S3 jvi.00706-25-s0003.pdf Representative quantification for the fold change of adaptor proteins during virus infection.

Fig. S4 jvi.00706-25-s0004.pdf Representative quantification for the fold change of adaptor proteins during virus infection.

Fig. S5 jvi.00706-25-s0005.pdf Taurine did not affect the activation of the NF-κB canonical pathway.

Fig. S6 jvi.00706-25-s0006.pdf Representative quantification for the fold change of adaptor proteins during virus infection.

Fig. S7 jvi.00706-25-s0007.pdf Representative quantification for the fold change of adaptor proteins during virus infection.

Fig. S8 jvi.00706-25-s0008.pdf Representative quantification for the fold change of adaptor proteins after stimulations.

ASM does not own the copyrights to Supplemental Material that may be linked to, or accessed through, an article. The authors have granted ASM a non-exclusive, world-wide license to publish the Supplemental Material files. Please contact the corresponding author directly for reuse.
