# Supplementary material for: CSAD inhibits excessive inflammation during viral infections through the NF-κB signaling pathway
Source: J Virol. 2025 Sep 15;99(10):e00706-25. doi: 10.1128/jvi.00706-25 (PMC12548428; doi:10.1128/jvi.00706-25)
Supplement: Fig. S1 — Taurine did not affect CSAD KO mice phenotype after PR8 infection. [file jvi.00706-25-s0001.pdf]

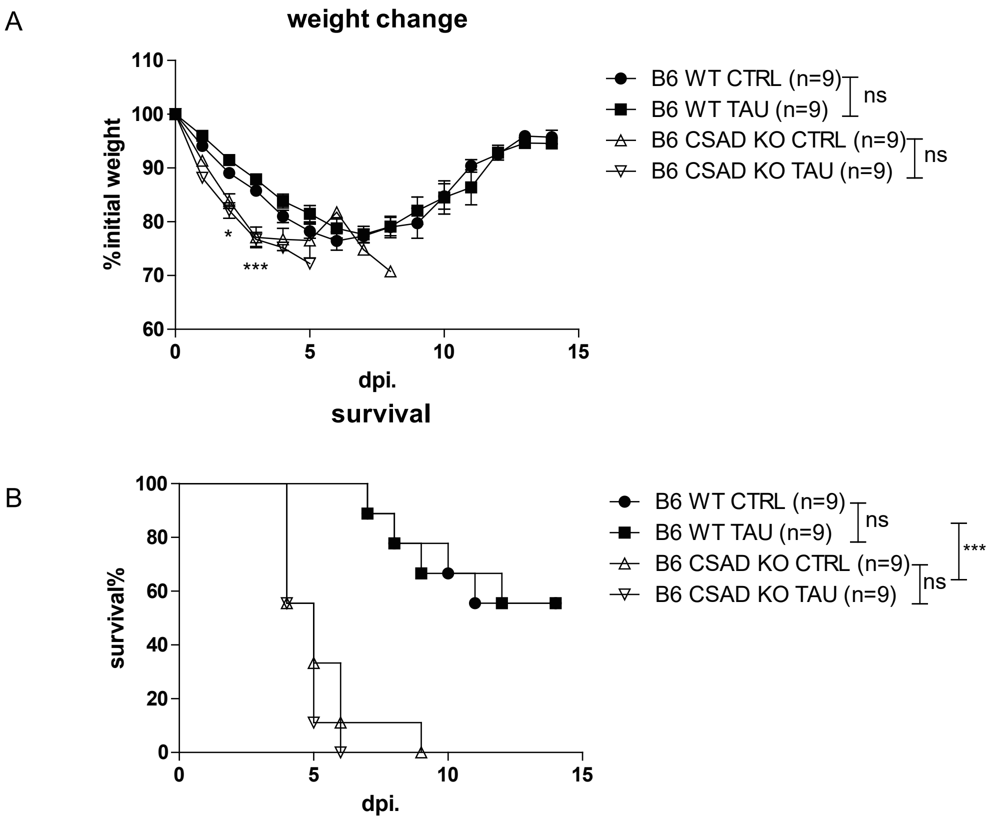

Fig S1. Taurine did not affect CSAD KO mice phenotype after PR8 infection. Weight change and survival rate of CSAD KO and B6 WT mice treated or not with taurine. A group of CSAD KO mice was continually treated with taurine daily after weaning. A group of aged-matched WT mice was treated with taurine 10 days before infection. Other groups of CSAD KO mice and WT mice did not receive taurine treatment. All the groups of mice were infected with 1×10^6 pfu PR8. In the taurine groups, CSAD KO or WT mice were continually treated with taurine till 15 dpi. The weight change (A) and survival rate (B) in all four groups of mice were monitored. The data correspond to the means ± SEM of one representative experiment from two similar independent experiments. *p < 0.05, **p < 0.01; ***p<0.001.
