# Supplementary material for: CSAD inhibits excessive inflammation during viral infections through the NF-κB signaling pathway
Source: J Virol. 2025 Sep 15;99(10):e00706-25. doi: 10.1128/jvi.00706-25 (PMC12548428; doi:10.1128/jvi.00706-25)
Supplement: Fig. S2 — CSAD is widely expressed in human and mouse tissues and organs. [file jvi.00706-25-s0002.pdf]

A

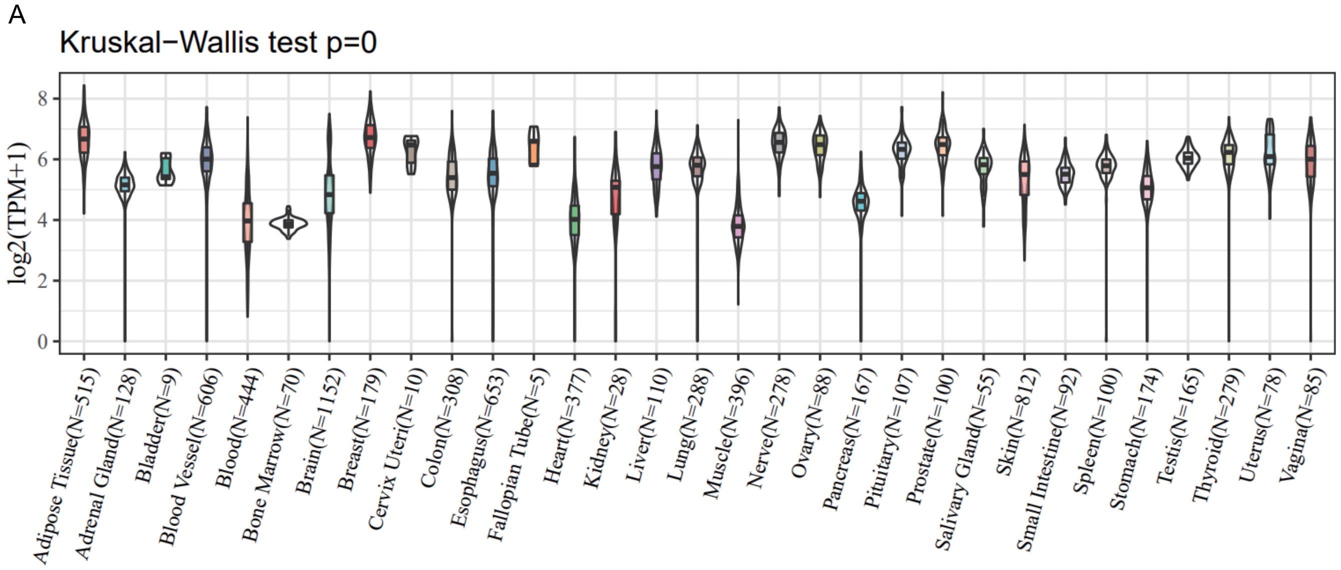

B

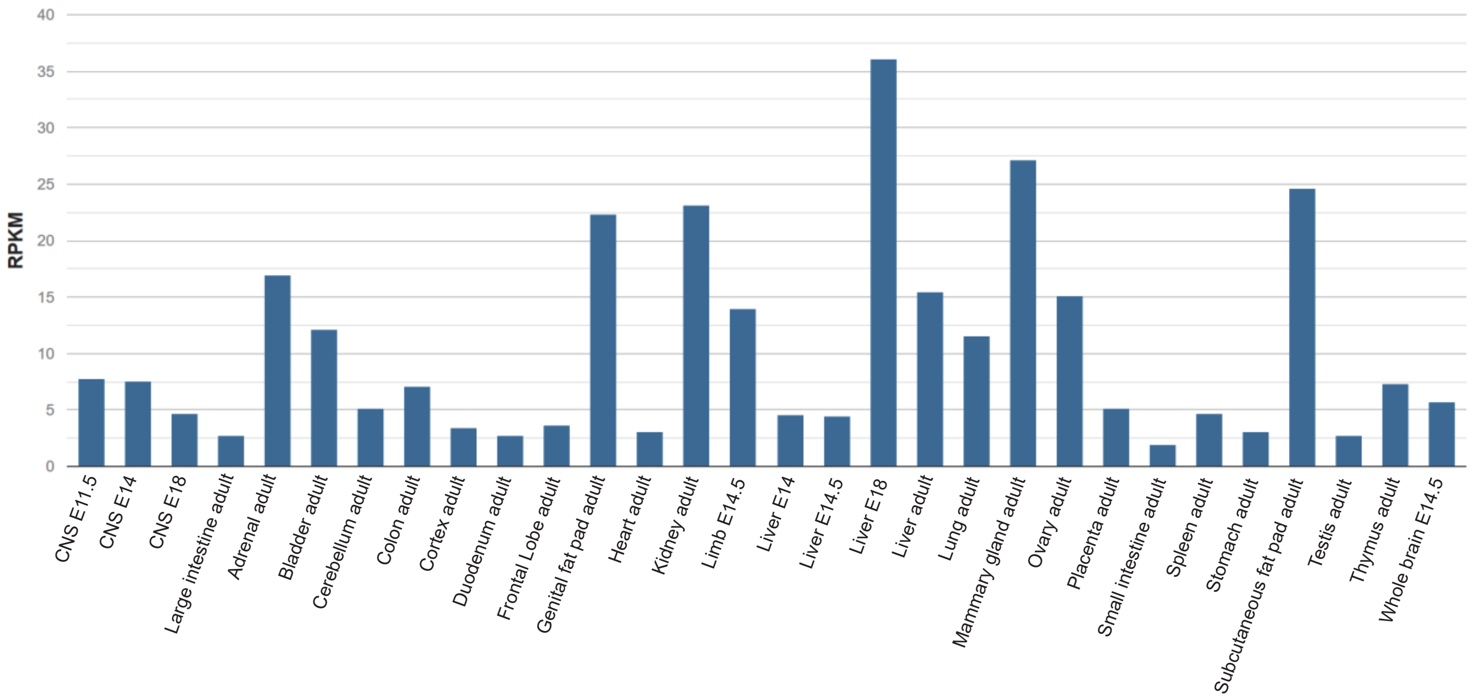

Fig S2. CSAD is widely expressed in human and mouse tissues and organs. (A) The expression levels of CSAD in different normal human tissues and organs. GTEx (Genotype-Tissue Expression) database was used to analyze the expression of CSAD in different normal human tissues and organs. The data visualization is displayed through the SangerBox data analysis platform. (B) Transcription levels of CSAD in different mouse tissues and organs. The information on CSAD transcription levels in different mouse tissues was obtained from NCBI. Data available at the website: https://www.ncbi.nlm.nih.gov/gene/246277/?report=expression
