# Supplementary material for: CSAD inhibits excessive inflammation during viral infections through the NF-κB signaling pathway
Source: J Virol. 2025 Sep 15;99(10):e00706-25. doi: 10.1128/jvi.00706-25 (PMC12548428; doi:10.1128/jvi.00706-25)
Supplement: Fig. S3 — Representative quantification for the fold change of adaptor proteins during virus infection. [file jvi.00706-25-s0003.pdf]

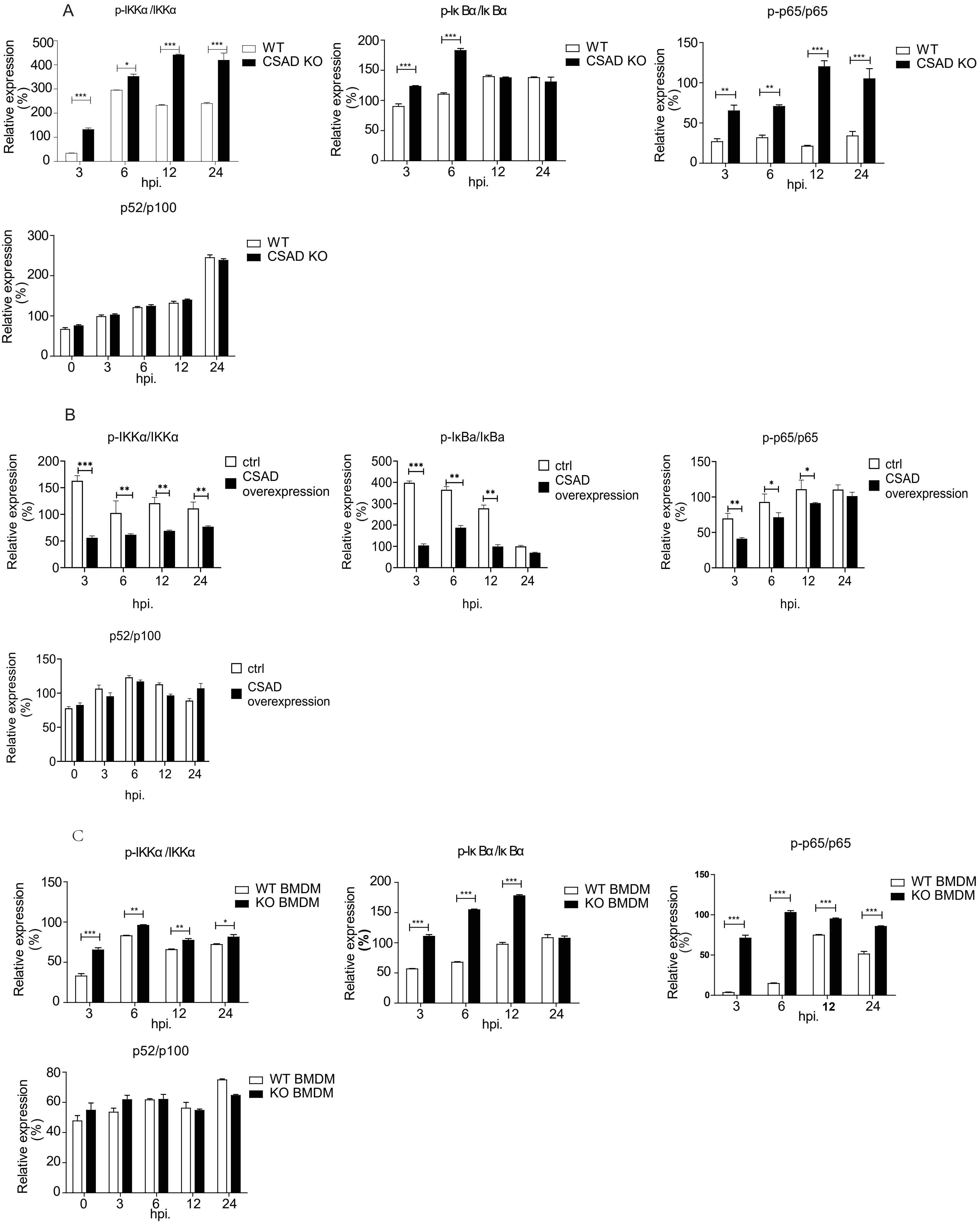

Fig S3. Representative quantification for the fold change of adaptor proteins during virus infection. (A) Quantification for the fold change of p-IKKα,p-IκBα,p-52,and p-p65 in WT and CSAD KO cells during PR8 infection, corresponding to Figure 4B.(B) Quantification forthe fold change of p-IKKα, p-IκBα,p-52,and p-p65 in 293T ctrl and CSAD overexpression cells during PR8 infection, corresponding to Figure 4C.(C) Quantification for the fold change of p-IKKα, p-IκBα,p-52,and p-p65 in B6 WT and CSAD KO mice BMDM during PR8 infection, corresponding to Figure 4D. Bands were quantified by TANON GIS software and the relative expression was calculated and analyzed.TANON GIS software and the relative expression was calculated and analyzed. Data are presented as the mean ± SEM from three independent experiments. *,p<0.05; **, p<0.01; ***, p<0.001.
