# Supplementary material for: CSAD inhibits excessive inflammation during viral infections through the NF-κB signaling pathway
Source: J Virol. 2025 Sep 15;99(10):e00706-25. doi: 10.1128/jvi.00706-25 (PMC12548428; doi:10.1128/jvi.00706-25)
Supplement: Fig. S5 — Taurine did not affect the activation of the NF-κB canonical pathway. [file jvi.00706-25-s0005.pdf]

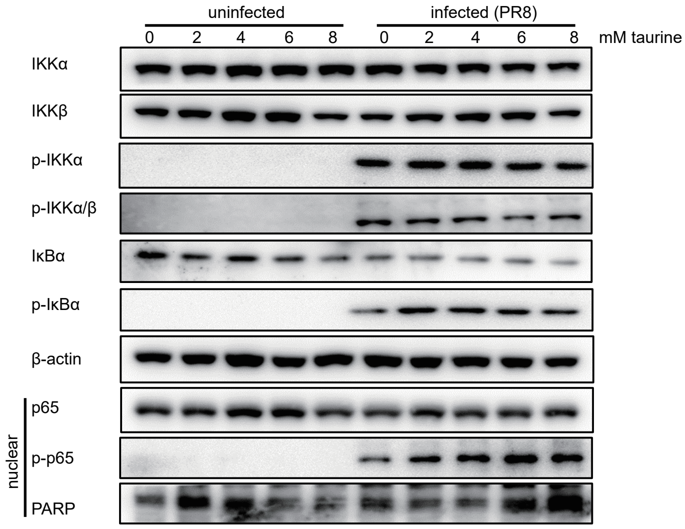

Fig S5. Taurine did not affect the activation of the NF-κB canonical pathway. Cells were treated with different concentrations of taurine (0, 2, 4, 6, 8 mM), and then were infected with PR8 at MOI=1. Cells were lysed at 6 hpi., and the levels of IKKα, IKKβ, p-IKKα, p-IKKα/β, IκB-α, p-IκB-α CSAD, and β-actin in total cell lysates, and p65, p-p65 or PARP in nuclear lysates were measured by western blot assay with the indicated antibodies. The data correspond to one representative experiment from at least three independent experiments.
