# Supplementary material for: CSAD inhibits excessive inflammation during viral infections through the NF-κB signaling pathway
Source: J Virol. 2025 Sep 15;99(10):e00706-25. doi: 10.1128/jvi.00706-25 (PMC12548428; doi:10.1128/jvi.00706-25)
Supplement: Fig. S6 — Representative quantification for the fold change of adaptor proteins during virus infection. [file jvi.00706-25-s0006.pdf]

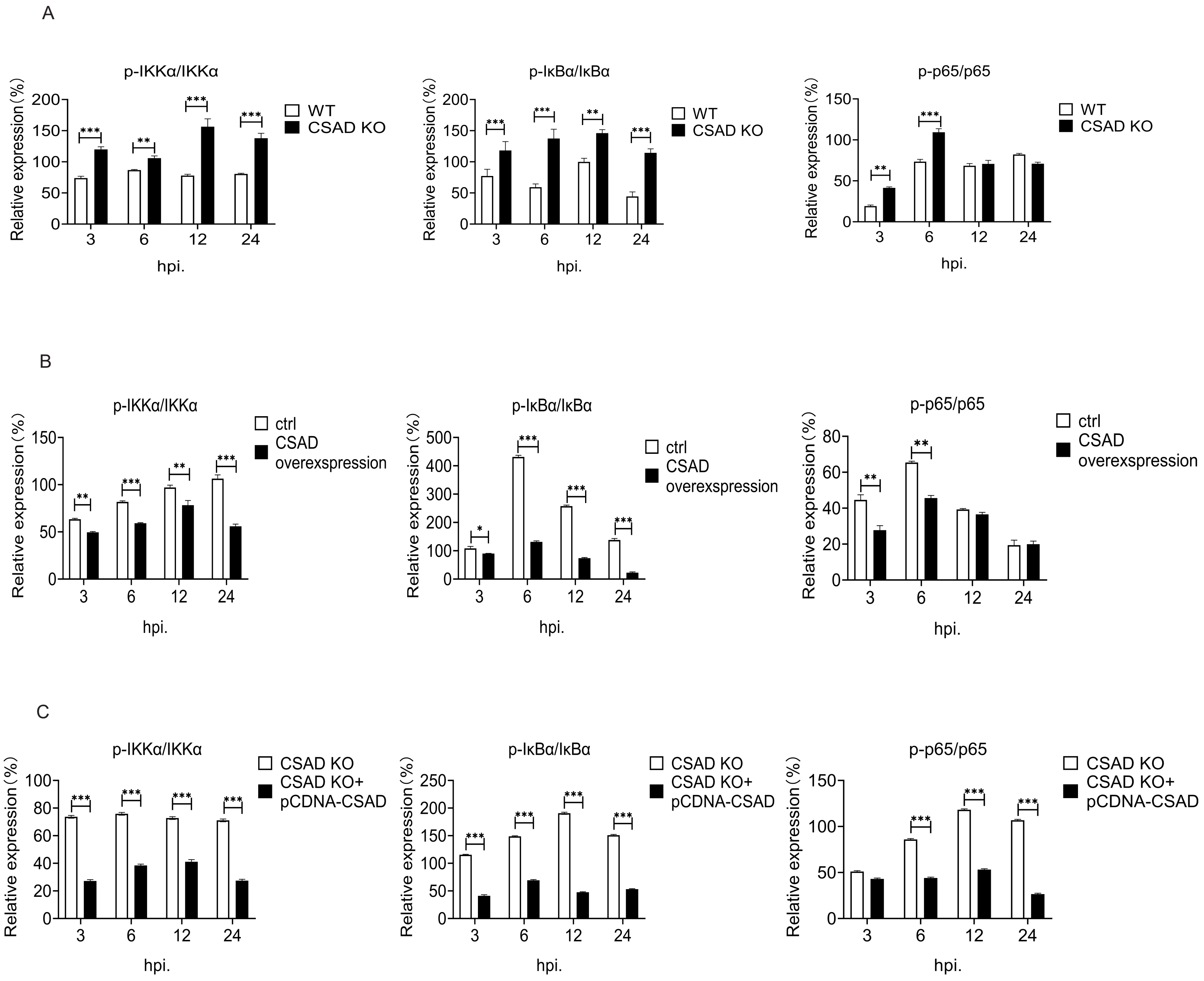

Fig S6. Representative quantification for the fold change of adaptor proteins during virus infection. (A) Quantification for the fold change of p-IKKα,p-IκBα,and p-p65 in WT and CSAD KO cells during CA0001 infection, corresponding to Figure 6A.(B) Quantification for the fold change of p-IKKα, p-IκBα,and p-p65 in 293T ctrl and CSAD overexpression cells during CA0001 infection, corresponding to Figure 6B.(C) Quantification for the fold change of p-IKKα, p-IκBα,and p-p65 in 293T CSAD KO and CSAD KO+pCDNA-CSAD cells during CA0001 infection, corresponding to Figure 6C. Bands were quantified by TANON GIS software and the relative expression was calculated and analyzed.TANON GIS software and the relative expression was calculated and analyzed. Data are presented as the mean ± SEM from three independent experiments. *,p<0.05; **, p<0.01; ***, p<0.001.
