# Supplementary material for: CSAD inhibits excessive inflammation during viral infections through the NF-κB signaling pathway
Source: J Virol. 2025 Sep 15;99(10):e00706-25. doi: 10.1128/jvi.00706-25 (PMC12548428; doi:10.1128/jvi.00706-25)
Supplement: Fig. S7 — Representative quantification for the fold change of adaptor proteins during virus infection. [file jvi.00706-25-s0007.pdf]

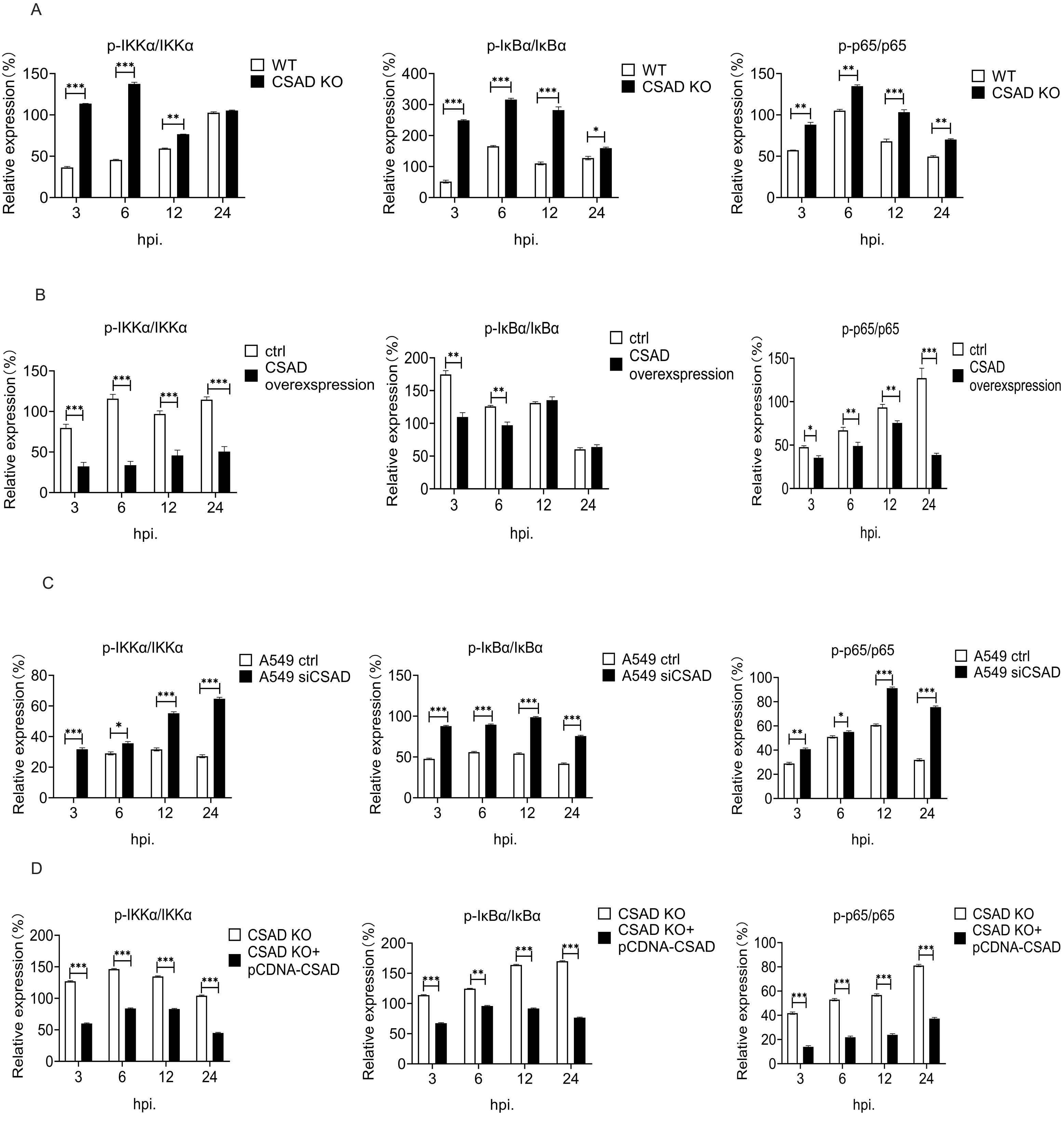

Fig S7. Representative quantification for the fold change of adaptor proteins during virus infection. (A) Quantification for the fold change of p-IKKα,p-IκBα, and p-p65 in WT and CSAD KO cells during VSV infection, corresponding to Figure 6D.(B) Quantification for the fold change of p-IKKα, p-IκBα,and p-p65 in 293T ctrl and CSAD overexpression cells during VSV infection, corresponding to Figure 6E.(C) Quantification for the fold change of p-IKKα, p-IκBα,and p-p65 in A549 ctrl and A549 siCSAD cell during VSV infection, corresponding to Figure 6F. (D)Quantification for the fold change of p-IKKα, p-IκBα,and p-p65 in 293T CSAD KO and CSAD KO+pCDNA-CSAD cells during VSV infection, corresponding to Figure 6G. Bands were quantified by TANON GIS software and the relative expression was calculated and analyzed.TANON GIS software and the relative expression was calculated and analyzed. Data are presented as the mean ± SEM from three independent experiments. *,$p<0.05$; **, $p<0.01$; ***, $p<0.001$.
