# Supplementary material for: CSAD inhibits excessive inflammation during viral infections through the NF-κB signaling pathway
Source: J Virol. 2025 Sep 15;99(10):e00706-25. doi: 10.1128/jvi.00706-25 (PMC12548428; doi:10.1128/jvi.00706-25)
Supplement: Fig. S8 — Representative quantification for the fold change of adaptor proteins after stimulations. [file jvi.00706-25-s0008.pdf]

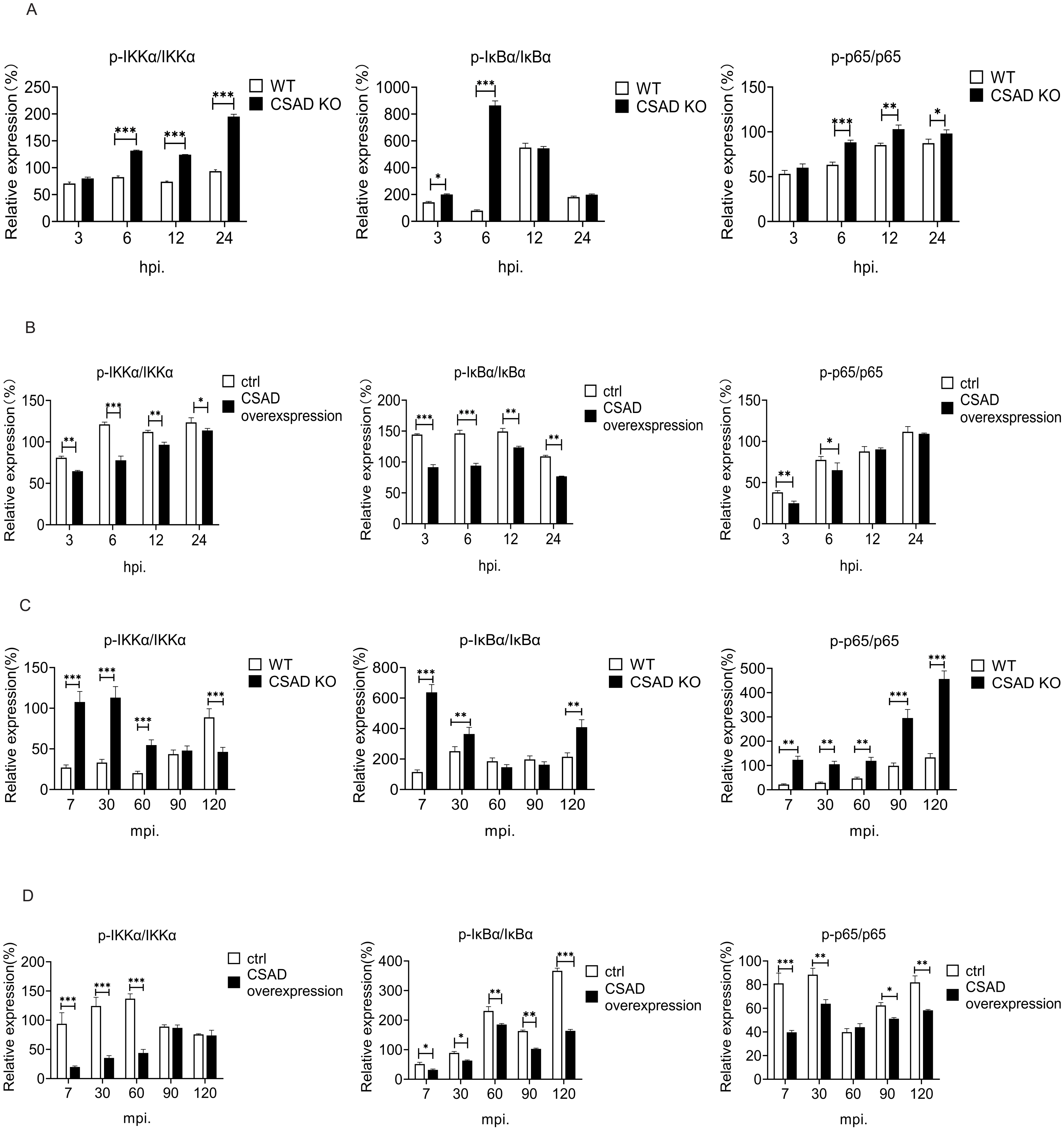

Fig S8. Representative quantification for the fold change of adaptor proteins after stimulations. (A) Quantification for the fold change of p-IKKα,p-IκBα,and p-p65 in WT and CSAD KO cells during polyI:C infection, corresponding to Figure 7A.(B) Quantification for the fold change of p-IKKα, p-IκBα,and p-p65 in 293T ctrl and CSAD overexpression cells during polyI:C infection, corresponding to Figure 7B.(C) Quantification for the fold change of p-IKKα, p-IκBα,and p-p65 in WT and CSAD KO cells during TNF-α infection, corresponding to Figure 7C.(D) Quantification for the fold change of p-IKKα, p-IκBα,and p-p65 in 293T ctrl and CSAD overexpression cells during TNF-α infection, corresponding to Figure 7D. Bands were quantified by TANON GIS software and the relative expression was calculated and analyzed.TANON GIS software and the relative expression was calculated and analyzed. Data are presented as the mean ± SEM from three independent experiments. *,$p<0.05$; **, $p<0.01$; ***, $p<0.001$.
